# Role of α-Catenin and its mechanosensing properties in regulating Hippo/YAP-dependent tissue growth

**DOI:** 10.1371/journal.pgen.1008454

**Published:** 2019-11-07

**Authors:** Ritu Sarpal, Victoria Yan, Lidia Kazakova, Luka Sheppard, Jessica C. Yu, Rodrigo Fernandez-Gonzalez, Ulrich Tepass

**Affiliations:** 1 Department of Cell and Systems Biology, University of Toronto, Toronto, Ontario, Canada; 2 Institute of Biomaterials and Biomedical Engineering, University of Toronto, Toronto, Ontario, Canada; 3 Developmental and Stem Cell Biology Program, The Hospital for Sick Children, Toronto, Ontario, Canada; University of North Carolina at Chapel Hill, UNITED STATES

## Abstract

α-catenin is a key protein of adherens junctions (AJs) with mechanosensory properties. It also acts as a tumor suppressor that limits tissue growth. Here we analyzed the function of Drosophila α-Catenin (α-Cat) in growth regulation of the wing epithelium. We found that different α-Cat levels led to a differential activation of Hippo/Yorkie or JNK signaling causing tissue overgrowth or degeneration, respectively. α-Cat can modulate Yorkie-dependent tissue growth through recruitment of Ajuba, a negative regulator of Hippo signaling to AJs but also through a mechanism independent of Ajuba recruitment to AJs. Both mechanosensory regions of α-Cat, the M region and the actin-binding domain (ABD), contribute to growth regulation. Whereas M is dispensable for α-Cat function in the wing, individual M domains (M1, M2, M3) have opposing effects on growth regulation. In particular, M1 limits Ajuba recruitment. Loss of M1 causes Ajuba hyper-recruitment to AJs, promoting tissue-tension independent overgrowth. Although M1 binds Vinculin, Vinculin is not responsible for this effect. Moreover, disruption of mechanosensing of the α-Cat ABD affects tissue growth, with enhanced actin interactions stabilizing junctions and leading to tissue overgrowth. Together, our findings indicate that α-Cat acts through multiple mechanisms to control tissue growth, including regulation of AJ stability, mechanosensitive Ajuba recruitment, and dynamic direct F-actin interactions.

## Author summary

We explore the regulation of tissue and organ size which is an important consideration in normal development and health. During development, tissues reach specific sizes in proportion to the rest of the body. Uncontrolled growth can lead to malformations or promote tumor growth. Recent findings have emphasized an important role for mechanical cues in the regulation of tissue growth. Mechanical signals can, for example, arise from cytoskeletal contraction that increases tension, or from compression due to proliferation and a resulting increase in cell density that would lower tension. Mechanosensory molecules that are sensitive to changes in tissue tension can convert mechanical cues into biochemical signals that enhance or slow proliferation or cell death to adjust overall tissue size. One such mechanosensory molecule is α-Catenin which is a key component of cell adhesion structures that physically link cells together and couples these structures to the cytoskeleton within cells. We clarify several molecular parameters of how α-Catenin regulates signalling pathways that control cell proliferation and cell death.

## Introduction

The cadherin-catenin complex (CCC) at adherens junctions (AJs) links actomyosin networks of neighboring cells thereby sensing and distributing cytoskeletal tension across tissues [1, 2]. α-catenin physically couples the cadherin-β-catenin complex to the actin cytoskeleton. Loss of α-catenin function, similar to the loss of E-cadherin, disrupts epithelial integrity including a loss of apical-basal polarity, cell adhesion, and the ability of cells to undergo coordinated cell movements. α-catenin can also act as a mechanosensor. Biochemical responses to mechanosensing are thought to have multiple consequences [3, 4, 5], including a strengthening of F-actin binding to enhance cell adhesion [6–9], reorganization of actin at cell junctions [9], and modulation of cell signalling that regulates tissue growth through the Hippo/YAP pathway [10]. However, in vivo evidence for specific functions of α-catenin-mediated mechanosensing remains limited.

E-cadherin and α-catenin act as tumor suppressor proteins [11–13]. Deregulation of the transcriptional co-activator YAP (Yorkie [Yki] in Drosophila) is one mechanism of how the loss of the CCC can impact tissue growth. YAP is the key effector of the Hippo-Warts kinase cascade (MST1/2 and LATS1/2 in mammals) [14, 15]. α-catenin can regulate YAP/Yki through multiple, potentially cell type-specific mechanisms including cytoplasmic sequestration or activation of an integrin-Src pathway [16–18]. Moreover, active Yki not only acts in the nucleus to stimulate growth but is also recruited to apical junctions where it enhances myosin II activity, which in turn promotes tissue growth through the Hippo/Yki pathway [19].

It was proposed recently that mechanosensing of Drosophila α-Catenin (α-Cat) can modulate the Hippo/Yki pathway via the LIM domain protein Ajuba (Jub), a negative regulator of Warts (Wts). In response to cytoskeletal tension, α-Cat sequesters a Jub-Wts complex at AJs, preventing the phosphorylation of Yki and causing tissue growth [10, 20, 21]. A similar AJ-dependent mechanism appears to be conserved in mammals [14, 15]. However, loss of function of α-Cat, DE-cadherin (DEcad), and the Drosophila β-catenin protein, Armadillo (Arm), were reported to have a negative impact on growth and caused reduced Yki activity in mutant cells and enhanced JNK signaling leading to cell death [22]. Here, we address these discrepancies and present evidence suggesting that depending on the degree of α-Cat loss a differential activation of the Hippo/Yki and JNK pathways is observed, which causes either tissue overgrowth driven by Yki activation or tissue undergrowth driven by JNK-elicited cell death.

α-catenin has mechanosensory properties. α-catenin comprises three evolutionary conserved regions: a N-terminal region that binds to β-catenin, a central M region, and a C-terminal actin-binding domain (ABD). The activity of cytoplasmic myosin II applies tension to α-catenin when bound to β-catenin and F-actin, causing at least two conformational changes: First, the N-terminal α-helix within the ABD (α1-helix) is pulled away from ABD revealing an enhanced F-actin binding interface and facilitating dimerization of ABD to promote actin bundling [9]. This mechanosensory property of α-catenin ABD explains its catch bond behavior, the ability of a chemical bond to strengthen as a result of force application [7]. Second, the M region of α-catenin also undergoes a conformational change when stretched. M consists of three α-helical bundles, the M1, M2, and M3 domains. The angle between the M2 and M3 domains increases upon force application and the M1 domain unfurls to reveal a cryptic binding site for the actin-binding protein Vinculin [6, 8, 23–25]. The recruitment of Vinculin is thought to strengthen the AJ-actin link. However, in vivo evidence supporting this hypothesis is lacking.

The Drosophila wing disc epithelium has been a crucial model to investigate the regulation of tissue growth [26]. The JNK and Hippo/Yki pathways monitor epithelial health and play key roles in the regulation of disc size. Tissue growth in the wing epithelium is also influenced by mechanical tension [27]. Differences in tension correlate with Yki activation, a response thought to be mediated by AJ mechanosensing and the Hippo pathway [10, 21, 28]. We have tested this model by replacing endogenous α-Cat with mutant forms that compromise α-Cat M region or ABD-based mechanosensing, and asked how these α-Cat mutant isoforms can affect AJ stability, Jub recruitment and tissue growth.

## Results

### α-Catenin function in growth regulation and epithelial maintenance can be genetically separated

**Fig 1:**
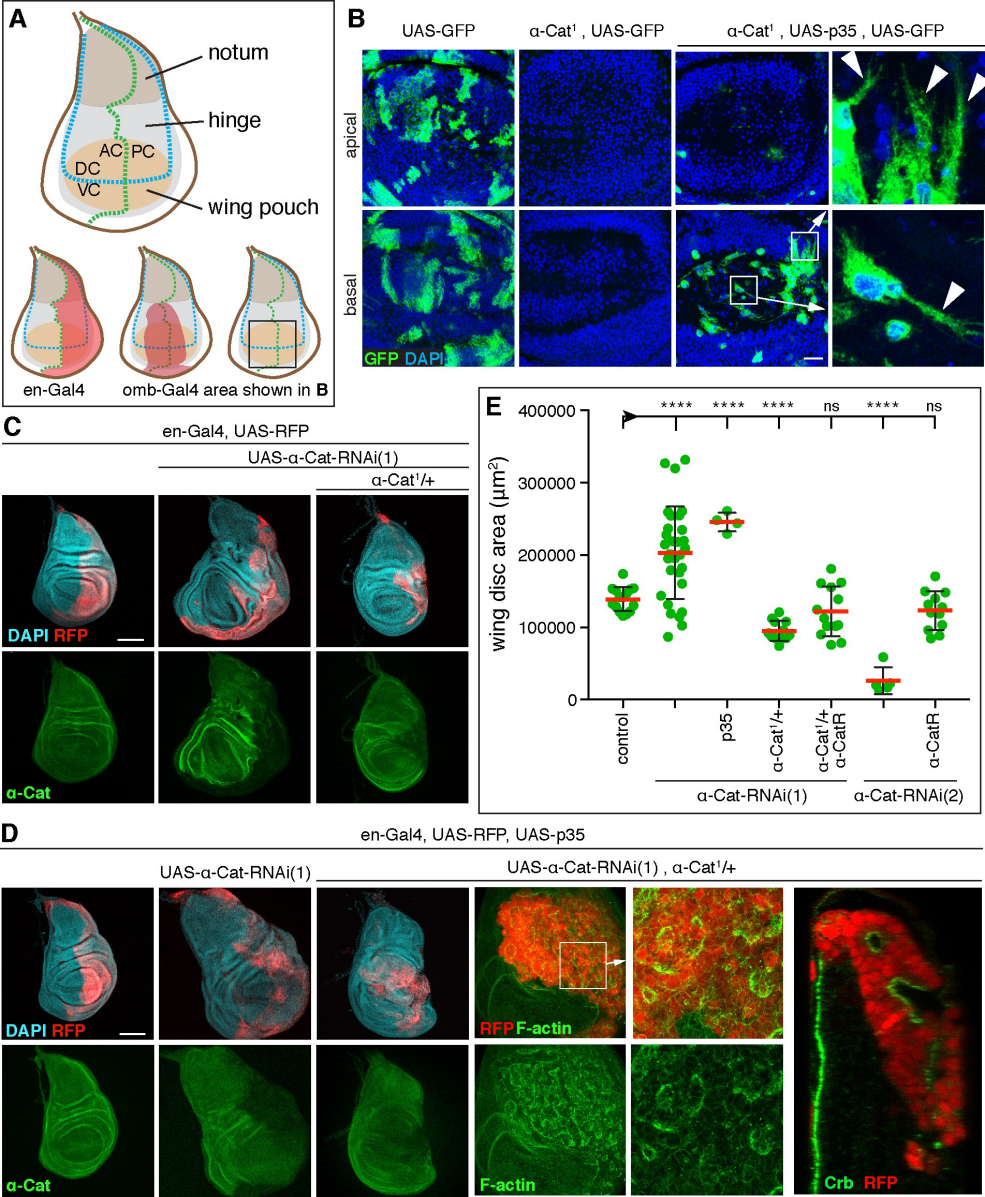
A phenotypic series for α-Cat reveals distinct roles in growth regulation and epithelial polarity. (A) Schematic of 3^rd^ larval instar wing imaginal disc. Indicated are the main subdivisions of the disc proper, the compartment boundaries (AC, anterior compartment; PC posterior compartment; DC, dorsal compartment; VC, ventral compartment), the expression domains of the *en-Gal4* and *omb-Gal4* drivers used in this study, and the area of the discs shown in (B). (B) Late 3^rd^ larval instar wing discs with control (left two panels) or *α-Cat*^*1*^ null mutant clones positively labeled with GFP. In contrast to control clones, *α-Cat*^*1*^ mutant clones are not observed. When cell death is suppressed through expression of p35, *α-Cat*^*1*^ mutant cells are found basal to the epithelium and show extensive protrusive activity (arrowheads in close-ups). Scale bar, 25 μm. (C) KD of α-Cat in the PC (marked by RFP) with *α-Cat-RNAi(1)* causes tissue overgrowth, whereas KD of α-Cat in the PC with *α-Cat-RNAi(1)* in the presence of one copy of *α-Cat*^*1*^ causes a degeneration of the PC. Scale bar, 100 μm. (D) KD of α-Cat in the PC (marked by RFP) with *α-Cat-RNAi(1)* while expressing p35 causes tissue overgrowth, whereas KD of α-Cat in the PC with *α-Cat-RNAi(1)* while expressing p35 in the presence of one copy of *α-Cat*^*1*^ causes the formation of a multilayered tumor mass with small epithelial vesicles or patches. Apical domain of epithelial cells marked by enrichment of F-actin and Crb. Scale bars, 100 μm. (E) Quantification of wing disc area in flies of indicated genotypes. Two-tailed, unpaired t-test used to determine statistical significance. ns (P>0.05), ****(P≤0.0001).

Our previous work suggested that the concentration of the CCC can be reduced to a few percent of normal levels and still maintain normal AJs and epithelial integrity in tissues that do not undergo cell rearrangements such as the late embryonic epidermis [29, 30]. To test whether α-Cat has a cell-autonomous function in growth regulation we took advantage of the wing disc (Fig 1A), which shows dramatic tissue growth but little cell rearrangement during larval development. Based on findings in the embryo, we reasoned that a moderate reduction of α-Cat would not disrupt epithelial polarity and cause cell death in the developing wing. In such a scenario we could potentially reveal defects in tissue growth resulting from the partial loss of α-Cat. We examined tissue in which α-Cat was reduced to different degrees (a phenotypic series) starting with the null phenotype. Cell clones mutant for a null allele of *α-Cat* were not detected in the third larval wing imaginal disc in contrast to control clones, (Fig 1A and 1B) [30]. We expected that the loss of AJs initiates programmed cell death [22, 31]. Upon expression of the caspase inhibitor p35 [32] we found that *α-Cat* null cells accumulate below the disc epithelium and display spindle-shaped morphologies with extensive protrusions (Fig 1B). We conclude that wing epithelial cells devoid of α-Cat undergo programmed cell death, and if prevented from dying leave the epithelium and adopt a mesenchyme-like character.

To elicit a less drastic reduction of α-Cat we expressed two different *α-Cat* shRNAs in the posterior compartment (PC) of the wing disc with the *en-Gal4* driver (Fig 1A). *α-Cat-RNAi(1)* is directed against the 5’UTR of *α-Cat* whereas *α-Cat-RNAi(2)* targets a region in the *α-Cat* RNA that encodes the M2 domain. Expression of *α-Cat-RNAi(2)* led to a stronger knockdown (KD) of α-Cat than *α-Cat-RNAi(1)* (S1 Fig). *en>α-Cat-RNAi(1)* caused hyperplastic overgrowth of the wing disc with both enlarged anterior compartment (AC) and PC suggesting non-cell-autonomous and cell-autonomous tissue overgrowth (Fig 1C and 1E). Further reduction of α-Cat by expression of *α-Cat-RNAi(1)* in animals that carried one mutant copy of *α-Cat* (*en>α-Cat-RNAi(1)*, *α-Cat/+*) caused a reduction in disc size due to a degeneration of much of the PC (Fig 1C and 1E). An even more pronounced degeneration of the PC was seen upon expression of *α-Cat-RNAi(2)* (Fig 1E, and below), confirming that *α-Cat-RNAi(2)* causes a stronger KD of α-Cat than *α-Cat-RNAi(1)*. Notably, whereas the degeneration of the PC upon α-Cat KD has been reported previously [22], we found that a moderate KD of α-Cat caused a conspicuous tissue overgrowth, suggesting that α-Cat normally limits tissue growth.

We combined *α-Cat* KD with p35 expression to examine how cell death contributes to the KD phenotypes. *en>α-Cat-RNAi(1) p35* discs were larger compared to *en>α-Cat-RNAi(1)* discs (Fig 1D and 1E), suggesting that the hyperplastic discs resulting from moderate *α-Cat* KD showed a significant amount of cell death. Suppression of cell death in strong *α-Cat* KD conditions (*en>α-Cat-RNAi(1) p35*, *α-Cat/+*) led to cell-autonomous formation of multilayered tissue masses (Fig 1D). These tumor-like masses contained pockets of epithelial cells as was evident from labeling with the apical markers Crumbs (Crb) and F-actin (Fig 1D). Taken together, our analysis of *α-Cat* loss-of-function conditions in conjunction with a block of cell death defined three distinct phenotypic classes: (i) a moderate loss of α-Cat causes epithelial overgrowth, (ii) a strong reduction of α-Cat causes overgrowth associated with a partial loss of epithelial integrity, and (iii) complete removal of α-Cat causes a loss of epithelial integrity with cells showing protrusive activity.

### Differential activation of JNK and Hippo/Yki signaling in α-Cat compromised wing disc epithelia

**Fig 2:**
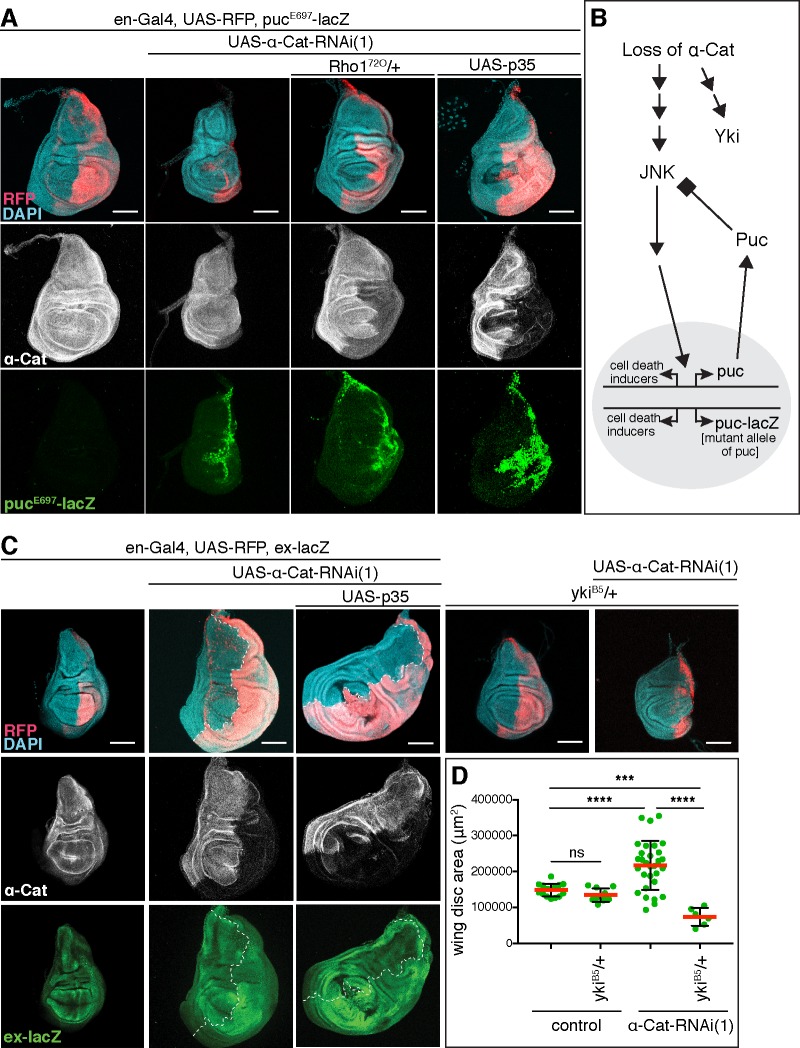
Loss of α-Cat activates JNK pathway and Yki. (A) Activation of the JNK transcriptional reporter *puc-LacZ* is seen when α-Cat is depleted in the PC. The strong reduction of the PC in *α-Cat-RNAi(1)* and *puc-LacZ* compared to *α-Cat-RNAi(1)* wing discs (see Fig 1C) indicates a genetic interaction between *α-Cat-RNAi(1)* and *puc-LacZ*. The loss of the PC can be rescued by expression of p35 or removal of one copy of Rho1. Scale bars, 100 μm. (B) Schematic illustrating the relationship between Puc and JNK. The phosphatase Puc dephosphorylates and therefore deactivates the JNK kinase. The presence of the *puc* mutant allele *puc-lacZ* is predicted to hyper-activate JNK in conjunction with a KD of α-Cat, which causes enhanced cell death as observed in (A). (C) Elevated expression of the Yki transcriptional reporter *ex-lacZ* is observed upon KD of α-Cat in the absence or presence of p35. Tissue overgrowth is suppressed through the addition of a null mutant copy of *yki* (*yki*^*B5*^). Scale bars, 100 μm. (D) Quantification of wing disc area in flies of indicated genotypes. Two-tailed, unpaired t-test; ns (P>0.05), ****(P≤0.0001), ***(P = 0.0003).

Activation of JNK signaling that causes cell death under strong *α-Cat* KD conditions was reported previously [22]. We confirmed these data. In addition, we tested whether JNK is activated with moderate *α-Cat* KD. Using the JNK transcriptional reporter *puc*^*E697*^*-lacZ (puc-lacZ)* we found that the JNK pathway was activated in *en>α-Cat-RNAi(1)* and *en>α-Cat-RNAi(1) p35* discs, in which cell death is blocked downstream of JNK activation through p35 expression (Fig 2A). Interestingly, *α-Cat-RNAi(1) puc-lacZ* discs did not show hyperplastic growth as observed for *α-Cat-RNAi(1)* but a strong degeneration of the PC. *puc-lacZ* is a loss of function allele of *puckered (puc)*, expected to cause higher JNK activity levels as Puc is a phosphatase that negatively regulates JNK signaling through dephosphorylation of the JNK kinase Basket (Fig 2B) [33, 34]. The genetic interaction between *α-Cat-RNAi(1)* and *puc-lacZ* is consistent with the notion that JNK signaling is activated in *α-Cat-RNAi(1)* discs. However, the levels of JNK-elicited cell death in *en>α-Cat-RNAi(1)* discs are apparently insufficient to overcome the concomitant tissue overgrowth resulting from the depletion of α-Cat.

p120, E-cadherin, and αE-catenin were recently shown to be haploinsufficient tumor suppressors in mammalian models for intestinal cancer [35]. Complete loss of mammalian p120 causes a disruption of the intestinal epithelium and cell death. The cell death observed upon the loss of p120 in intestinal cells that also lack the tumor suppressor APC is effectively blocked by inhibition of Rho kinase, suggesting that Rho1 signaling is required for apoptosis [35]. In Drosophila, the RhoGEF2-Rho1-Rho kinase-Myosin II pathway was linked to JNK activation [36, 37]. To determine if Rho1 was involved in activating JNK in *α-Cat-RNAi(1) puc-lacZ* wing discs we removed a copy of Rho1 in this background. Reduction of Rho1 levels suppressed cell death in *α-Cat-RNAi(1) puc-lacZ* discs (Fig 2A), suggesting that loss of α-Cat precipitates JNK activation through Rho1.

Given previous findings in mammalian cells, we anticipated that the activation of Yki is responsible for the cell-autonomous overgrowth seen in *α-Cat-RNAi(1)* discs. Indeed, we found that the Yki transcriptional reporter *expanded (ex)-lacZ* is upregulated in the PC of *en>α-Cat-RNAi(1)* discs in addition to the previously reported [22], non-cell-autonomous upregulation in the AC (Fig 2C). Moreover, combined partial reduction of α-Cat and Yki (*α-Cat-RNAi(1)*, *yki/+)* suppressed the *α-Cat-RNAi(1)* elicited overgrowth (Fig 2C and 2D). These findings indicate that a reduction of α-Cat causes a cell-autonomous upregulation of Yki activity. Collectively, our findings suggest that the response to the loss of α-Cat function is governed by the differential activation of Yki and JNK signaling with Yki activation being more sensitive, causing tissue overgrowth under moderate α-Cat KD conditions.

### α-Catenin acts as an adherens junction protein to limit growth

**Fig 3:**
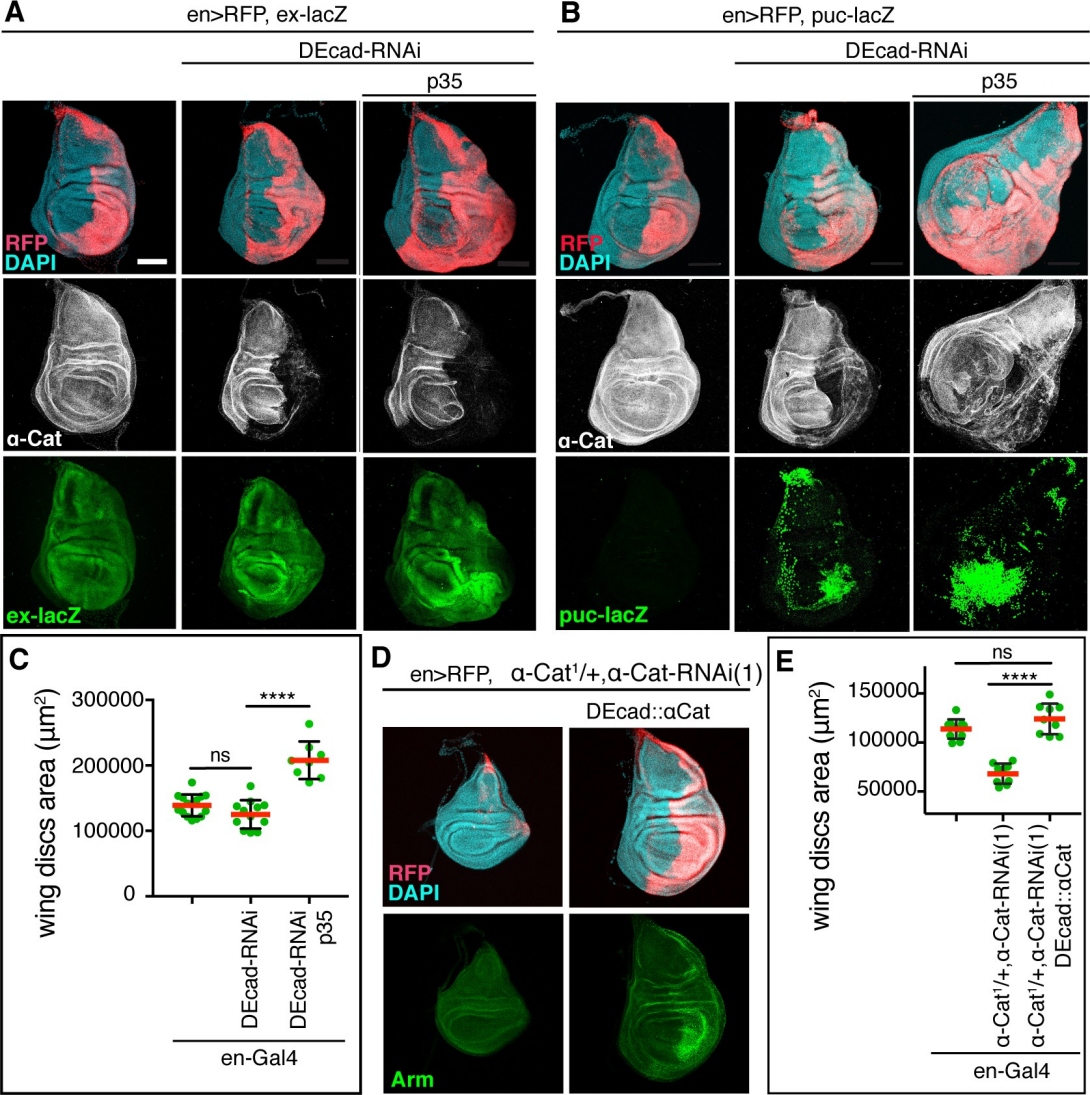
α-Cat regulates tissue growth as a DEcad binding partner. (A) KD of DEcad enhances activation of the Yki transcriptional reporter *ex-LacZ*. KD of DEcad in conjunction with expression of p35 causes tissue overgrowth. Scale bars, 100 μm. (B) KD of DEcad with or without co-expression of p35 causes activation of the JNK transcriptional reporter *puc-LacZ*. KD of DEcad in conjunction with expression of p35 causes tissue overgrowth. Scale bars, 100 μm. (C) Quantification of late 3^rd^ larval instar wing disc area in DEcad KD flies. Two-tailed, unpaired t-test; ns (P>0.05), ****(P≤0.0001). (D, E) Sample discs (D) and quantification of disc area (E) of late 3^rd^ larval instar wing discs of the indicated genotypes. Expression of DEcad::αCat in *en>α-Cat-RNAi(1)*, *α-Cat/+* restored normal wing disc size as seen in en-Gal4 controls. Two-tailed, unpaired t-test; ns (P>0.05), ****(P≤0.0001).

Several studies have suggested the possibility that mammalian αE-catenin has cadherin-independent functions, possibly also in the regulation of growth [reviewed in 2]. To address this question in Drosophila, we asked whether DEcad regulates tissue growth. We did not consider Arm as it is also a central player in the Wingless signaling pathway that is required for wing growth and development [38]. To reproduce the effect of a moderate α-Cat KD, we partially depleted DEcad with RNAi in a wild-type background or in the presence of caspase inhibitor p35. We found in both cases an enhanced activity of the Yki reporter *ex-lacZ* and the JNK reporter *puc-lacZ* (Fig 3A and 3B). Moreover, *en>DEcad-RNAi p35* flies showed enlarged wing discs similar to *en>α-Cat-RNAi(1)* or *en>α-Cat-RNAi(1) p35* (Fig 3C). These findings suggest that the concentration of the CCC plays an important role in growth regulation as the reduction of either α-Cat or DEcad could cause Yki activation and an overgrowth of the wing epithelium. To further address the question of whether α-Cat-based regulation of growth is mediated through its function as an AJ protein we expressed a DEcad::αCat fusion protein in *en>α-Cat-RNAi(1)*, *α-Cat/+* discs. This restored normal wing discs (Fig 3D and 3E) suggesting that DEcad::αCat can compensate for loss of α-Cat to maintain both epithelial polarity and normal tissue size. Together, our findings indicate that α-Cat regulates tissue growth as a core component of the CCC.

### The α-Cat N domain recruits Ajuba to adherens junctions

**Fig 4:**
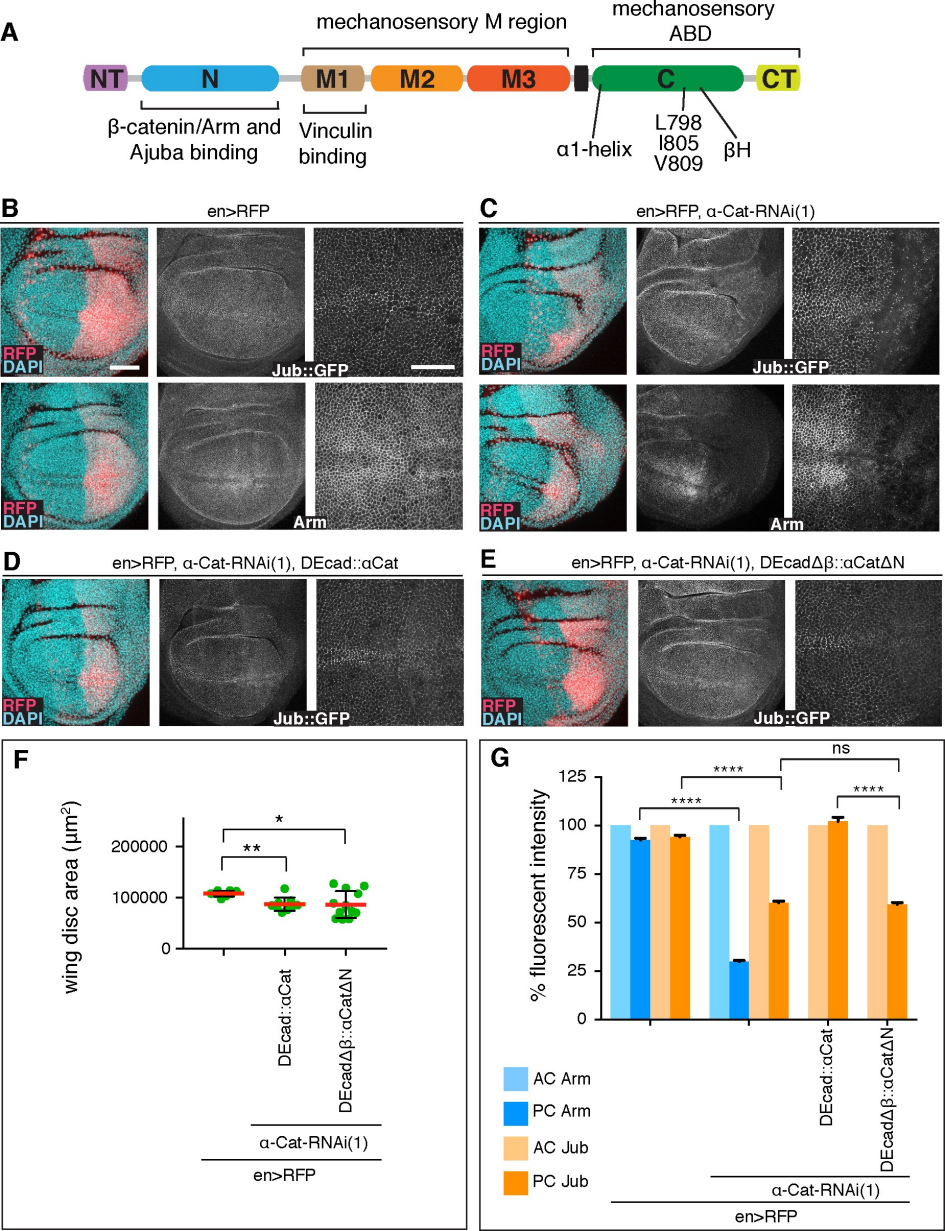
The α-Cat N domain recruits Jub to AJs. (A) α-Cat protein structure. (B) Control late 3^rd^ larval instar discs expressing *en-Gal4*, *UAS-RFP* and Jub::GFP, controlled by its endogenous promoter (upper panels), were stained for Arm (lower panels). Nuclei are labeled with DAPI. Close-up images to the right show wing pouch area on both sides of the anterior-posterior compartment boundary. Scale bars, 50 μm and 25 μm. (C) Same markers as in (A) with *UAS-α-CatRNAi(1)* expression in PC. (D) Late 3^rd^ larval instar discs expressing Jub::GFP, *en-Gal4*, *UAS-RFP*, *UAS-α-Cat-RNAi(1)*, and *UAS-DEcad*::*αCat*. (E) Late 3^rd^ larval instar discs expressing Jub::GFP, e*n-Gal4*, *UAS-RFP*, *UAS-α-Cat-RNAi(1)*, and *UAS-DEcadΔβ*::*αCatΔN*. (F) Quantification of wing disc area of flies of indicated genotypes. Two-tailed, unpaired t-test was used to determine statistical significance; *(P = 0.0180), **(P = 0.0012). (G) Comparison of relative fluorescent intensities between AC and PC for Jub::GFP and Arm. AC values were normalized to 100%. N = 200–500 cells from three wing discs. Mann Whitney test; ****(P≤0.0001), ns (P>0.05).

To explore the mechanism of how α-Cat contributes to growth regulation we examined its interaction with its binding partner Jub, an important regulator of Hippo signalling. The tension sensitive recruitment of Jub to AJs via its binding to α-Cat co-recruits Wts, which consequently can no longer phosphorylate Yki to inactivate it [10]. α-Cat is composed of a series of domains consisting of bundles of α-helices (Fig 4A). Similar to mammalian Ajuba, Drosophila Jub can bind to the N domain of α-Cat [39, 40]. We were therefore curious to determine whether the N domain is important for Jub recruitment to AJs in vivo and whether this recruitment is crucial for tissue growth. As the N domain also binds to Arm/β-catenin to couple α-Cat to the CCC, its removal from α-Cat renders α-Cat non-functional because it does not bind to the cadherin-β-catenin complex [41–43]. However, a fusion protein in which α-Cat lacking the N domain is fused to DEcadΔβ, which lacks the binding site for Arm/β-catenin (DEcadΔβ::αCatΔN) can functionally replace the endogenous complex (S2 Fig) and allowed us to test for the requirement of the N domain in growth regulation.

Monitoring Jub levels with Jub::GFP expressed under the control of its endogenous promoter [44], we found that Jub levels in the AC and PC of control discs are similar, whereas PC Jub::GFP levels were reduced to 60% in *en>a-Cat-RNAi(1)* KD discs. In addition, Arm concentration at AJs was reduced to 30% of normal (Fig 4B, 4C and 4G). *en>a-Cat-RNAi(1)* KD cells expressing DEcad::αCat (containing full-length α-Cat) restored normal Jub levels to AJs (Fig 4D and 4G). In contrast, junctional Jub levels remained at 60% in *en>a-Cat-RNAi(1)* KD cells expressing DEcadΔβ::αCatΔN similar to *en>a-Cat-RNAi(1)* cells (Fig 4E and 4G). Notably, the size of wing discs expressing either DEcad::αCat or DEcadΔβ::αCatΔN in *en>a-Cat-RNAi(1)* KD discs were similar (Fig 4F). These findings indicate that the N domain is essential for Jub recruitment to AJs but also that the α-Cat-mediated recruitment of Jub to AJs does not seem to have a linear relationship to growth regulation in the wing disc epithelium as normal growth is compatible with normal or low concentrations of Jub at AJs.

### α-Catenin overexpression causes Yki-mediated tissue overgrowth

**Fig 5:**
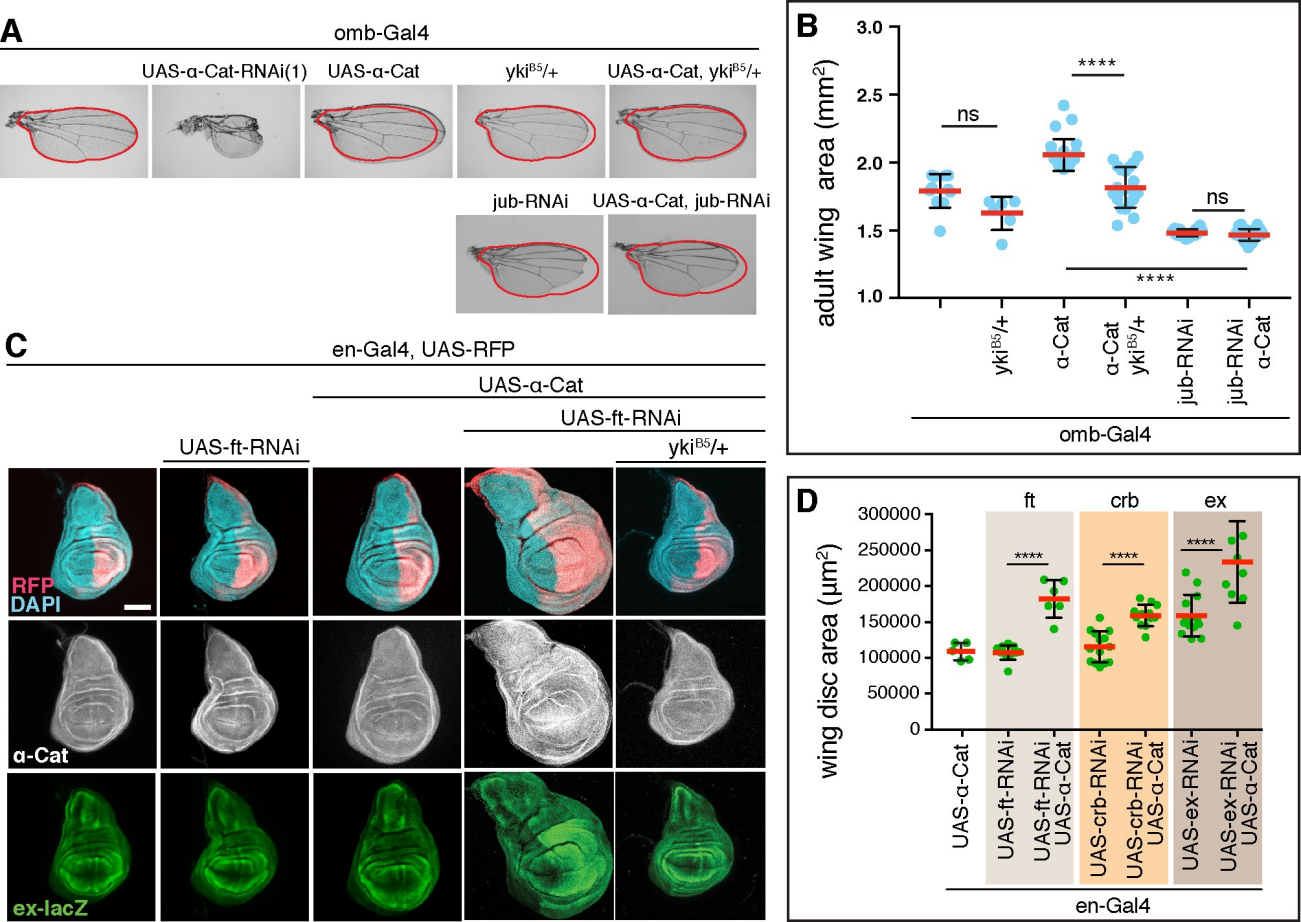
α-Cat overexpression activates Yki. (A, B) Overexpression of α-Cat causes an enlargement of the adult wing, which can be suppressed by one mutant copy of *yki*^*B5*^ or *jub-RNAi*. Two-tailed, unpaired t-test was used to determine statistical significance. ns(P>0.05), ****(P≤0.0001). (C, D) Overexpression of α-Cat does not cause a significant enlargement of the late 3^rd^ larval instar wing disc. However, overexpression of α-Cat in conjunction with a KD of an upstream regulator of the Hippo/Yki pathway (Fat, shown in (C), Crb and Ex shown in S3 Fig; all three interactions quantified in (D)) showed synergistic overgrowth phenotypes. Scale bars, 100 μm. Two-tailed, unpaired t-test; ****(P≤0.0001).

To further explore the relationship between α-Cat levels, Jub, and tissue growth we examined the effects of α-Cat overexpression. We found that α-Cat elicits wings with increased size when expressed with *omb-Gal4*, which is active in the central region of the developing wing pouch and hinge (Figs 1A, 5A and 5B). In contrast to the enlarged wings in *omb>α-Cat* flies, *omb>α-Cat-RNAi(1)* flies showed small wings with severe structural defects including notches and abnormal vein patterns, possibly the result of tissue degeneration at earlier stages of development (Fig 5A). Removing one copy of *yki* from *omb>α-Cat* flies or a knockdown of Jub normalized wing size (Fig 5A and 5B). These findings are consistent with the view that α-Cat overexpression activates Yki through a mechanism involving Jub.

We next examined larval wings discs overexpressing α-Cat with *en-Gal4* but did not detect any overgrowth (Fig 5C), suggesting that the observed increase in adult wing size emerges during pupal stages. Expression levels of our UAS-α-Cat construct are similar to endogenous α-Cat [9]. We reasoned therefore that the moderate overexpression of α-Cat alone may be insufficient to elicit excessive growth in a wild-type genetic background in larval wing discs. To reveal a potential impact of α-Cat overexpression on tissue growth in the disc, we therefore sensitized the genetic background by depletion of either Fat (Ft), Crb, or Ex, three positive upstream regulators of the Hippo pathway [45]. The shRNAs used to reduce Ft, Crb, and Ex caused little or no overgrowth on their own. However, when combined with α-Cat overexpression a synergistic overgrowth phenotype was observed in each case (Fig 5C and 5D; S3 Fig). For the interaction between α-Cat overexpression and *ft-RNAi* we also tested the effect on Yki activity and found an increase in the expression of the Yki reporter *ex-lacZ* and a suppression of the overgrowth and enhanced *ex-lacZ* expression when one copy of Yki was removed (Fig 5C). We conclude that α-Cat overexpression deregulates the Hippo/Yki pathway to cause overgrowth.

### The M region of α-Cat modulates tissue growth

α-Cat has mechanosensory properties [6, 9] which were proposed to direct wing growth through the tissue tension-dependent recruitment of Jub to AJs [10]. To assess the role of the two known mechanosensory domains of α-Cat, the M region and the ABD, in growth regulation, we used constructs that we published previously [9, 30, 43] and a new set of constructs that were rendered resistant to the both α-Cat shRNAs, *α-Cat-RNAi(1)* and *α-Cat-RNAi(2)*. These constructs were designated α-CatR, expression of wild-type protein, and α-CatR-XX, expression of mutant α-Cat isoforms. α-Cat and α-CatR rescued *α-Cat* mutants to adulthood [43; S2 Fig], and α-CatR fully rescued integrity and growth of *α-Cat-RNAi(1)* or *α-Cat-RNAi(2)* expressing wing epithelium (Fig 1E and see below). Expression levels of UAS-α-Cat is similar to endogenous α-Cat [9] whereas expression levels of UAS-α-CatR (which was generated in a different vector backbone) only reached ~2/3 of normal (63%; S4 Fig), although expressed from the same genomic insertion site as UAS-α-Cat (see Material and methods). α-CatR and α-CatR-XX proteins were effectively recruited to AJs in the wing disc epithelium (S5 Fig).

**Fig 6:**
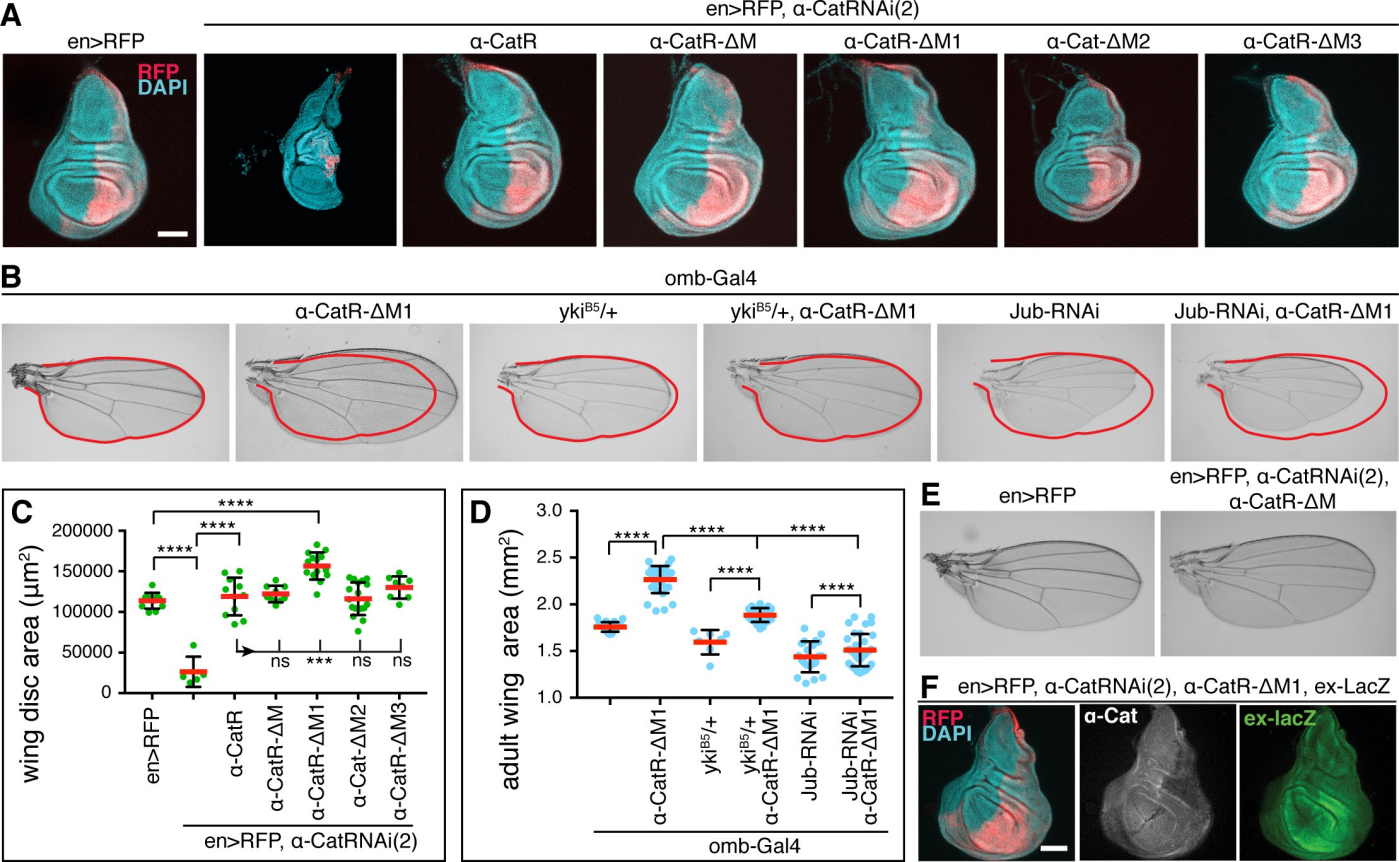
The M1 domain of α-Cat limits Jub- and Yki-dependent tissue overgrowth. (A) Late 3^rd^ larval instar wing discs of indicated genotypes labeled with DAPI and PC marked by RFP. α-Cat was depleted in PC with *α-Cat-RNAi(2)*. Scale bars, 100 μm. (B) Adult wings of flies of indicated genotypes. Both *yki*^*B5*^/+ and *jub-RNAi* suppress the overgrowth elicited by expression of α-CatR-ΔM1. (C) Quantification of late 3^rd^ larval instar wing disc area corresponding to (A) of flies of indicated genotypes. Two-tailed, unpaired t-test was used to determine statistical significance; ****(P≤0.0001), ***(P = 0.0003), ns (P>0.05). (D) Quantification of adult wing area corresponding to (B) of flies of indicated genotypes. Two-tailed, unpaired t-test was used to determine statistical significance; ****(P≤0.0001). (E) Wing of control (*en>RFP*) and *en>RFP*, *α-Cat-RNAi(2)*, *α-CatR-ΔM* adult fly. (F) Late 3^rd^ larval instar wing disc of the indicated genotype showing the failure of *α-CatR-ΔM1* to rescue tissue overgrowth in an α-Cat compromised background, and *ex-lacZ* expression remains increased compared to controls (see Fig 2C).

Mechanical tension can regulate tissue growth in the wing disc such that high tension elicits more growth than low tension [27, 46]. α-catenin is thought to respond to actomyosin generated tension through two conformational changes in the M region and the ABD, respectively. Tension reveals a cryptic binding site for the actin-binding protein Vinculin (Vinc) and perhaps other partners in the M region [6, 8, 47]. To test whether the M region plays a role in growth regulation, we deleted the entire M region from α-CatR (α-CatR-ΔM) and investigated *en>α-Cat-RNAi(2) α-CatR-ΔM* flies. The results were striking as α-CatR-ΔM was not only recruited to AJs in the wing epithelium (S5 Fig) but also fully restored a normal disc in both epithelial organization and size similar to α-CatR (Fig 6A and 6C). Moreover, flies survived to adulthood with normal wings (Fig 6E). These data show that the M region, and with it M region-based mechanosensing is not essential for growth regulation in the wing epithelium.

In striking contrast to *en>α-Cat-RNAi(2) α-CatR-ΔM* wing discs, *en>α-Cat-RNAi(2) α-CatR-ΔM1* wing discs showed hyperplastic overgrowth similar to discs with a moderate KD of α-Cat (Fig 6A and 6C), whereas *en>α-Cat-RNAi(2) α-Cat-ΔM2* and *en>α-Cat-RNAi(2) α-CatR-ΔM3* discs were normal in size (Fig 6A and 6C). Thus, all tested deletion constructs of the M region rescue epithelial polarity and growth defects of a strong α-Cat KD condition in the wing epithelium, with the exception of α-CatR-ΔM1 which rescued only epithelial polarity but not hyperplastic overgrowth. Consistently, we observed enhanced activity of the Yki reporter *ex-LacZ* in *en>α-Cat-RNAi(2) α-CatR-ΔM1* discs (Fig 6F). Moreover, in overexpression experiments, we found that α-CatR-ΔM1 elicited a stronger overgrowth in adult wings compared to α-CatR and other M region deletions of α-CatR (Fig 6B and 6D, S6 Fig). Removing one copy of *yki* in *omb>α-CatR-ΔM1* flies normalized adult wing size suggesting that overexpression of α-CatR-ΔM1 enhances Yki activity (Fig 6B and 6D). Collectively, our data suggest that M1 has an important role in limiting tissue growth.

### The M1 domain of α-Cat restricts the N-domain-dependent recruitment of Ajuba

The overgrowth caused by α-Cat-ΔM1 expression is suppressed by depletion of Jub (Fig 6B and 6D) raising the question whether Jub localization is affected in cells expressing M region deletion constructs. To address this question, we quantified the junctional concentration of Jub::GFP in AC control cells versus PC cells expressing *en>α-Cat-RNAi(2)* and an α-Cat construct and determined the concentration of the CCC by quantifying junctional Arm. The latter was done to find out whether potential changes in Jub levels reflect changes in CCC concentration given that Jub is a direct binding partner of α-Cat [10, 39, 40], and that this interaction is crucial for junctional localization of Jub (Fig 4). Alternatively, a lack of correlation in changes of junctional Jub and Arm would indicate that the tested α-Cat mutation affects the Jub-α-Cat interaction more directly.

**Fig 7:**
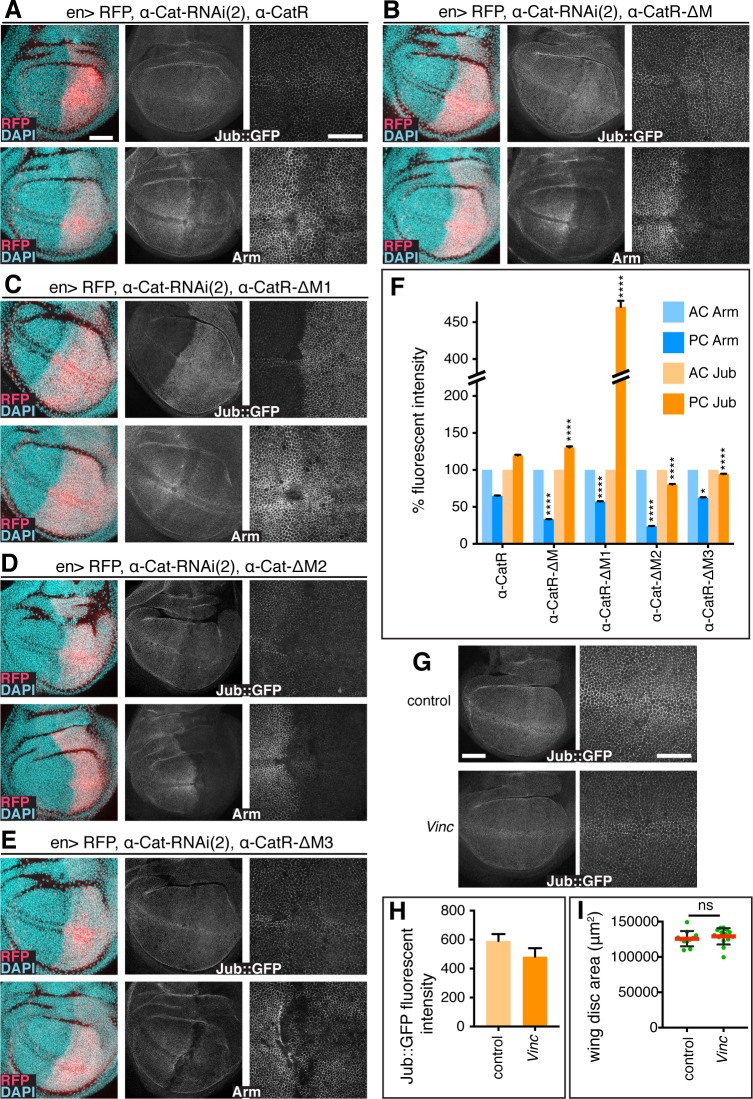
The M1 domain of α-Cat limits recruitment of Jub to AJs. (A-E) Late 3^rd^ larval instar discs expressing *en-Gal4*, *UAS-RFP*, *UAS-α-Cat-RNAi(2)* and either *UAS-α-CatR* (A), *UAS-α-Cat-ΔM* (B), *UAS-α-CatR-ΔM1* (C), *UAS-α-Cat-ΔM2* (D), or *UAS-α-CatR-ΔM3* (E). Discs express Jub::GFP controlled by its endogenous promoter (upper panels) and are stained for Arm (lower panels). Nuclei are labeled with DAPI. Close-up images to the right show wing pouch area on both sides of the anterior-posterior compartment boundary. Scale bars, 50 μm and 25 μm. (F) Comparison of relative fluorescent intensities between anterior compartment (AC) and posterior compartment (PC) for Jub::GFP (N = 500–1000 cells from five wing discs) and Arm (N = 500–1000 cells from four to six wing discs). AC values were normalized to 100%. Mann Whitney test was used to determine statistical significance comparing experimental and control (α-CatR) discs. ****(P≤0.0001), *(P = .0258). (G) Jub::GFP expression in control and *Vinc* null mutant (*Vinc*^*102*.*1*^*/Vinc*^*102*.*1*^) late 3^rd^ larval wing discs. (H) *vinc* null mutant and control late 3^rd^ larval wing discs have the same size. Two-tailed, unpaired t-test; ns (P>0.05) (I) Quantification of Jub::GFP fluorescent intensities (arbitrary units) in control and *vinc* null mutant discs (N>900 cells from five wing discs).

Arm levels were reduced to 66% of normal in *en>α-Cat-RNAi(2) α-CatR* control PC cells. Surprisingly, Jub::GFP was not reduced correspondingly but increased to 119% compared to AC cells (Fig 7A and 7F), indicating that junctional Jub does not directly follow CCC level, but that other mechanisms and possibly other binding partners could contribute to junctional localization of Jub. Similar to α-CatR, PC disc cells expressing M region deletions had lower Arm levels than seen in AC control cells. α-CatR-ΔM expressing cells showed only 32% of normal Arm levels but, interestingly, also a ~30% increase in junctional Jub::GFP (131%; Fig 7B and 7F). α-CatR-ΔM3 had Arm levels of 64% similar to α-CatR but Jub was not enhanced (98%; Fig 7E and 7F). α-Cat-ΔM2 had the lowest Arm levels with 22% and also showed a reduction of Jub::GFP to 78% (Fig 7D and 7F). This is consistent with our previous findings that the M2 region has a strong impact on junctional stability [43]. The most striking finding was the more than 4-fold increase (468% of normal) in junctional Jub::GFP levels observed with α-CatR-ΔM1 with Arm levels at only 59% (Fig 7C and 7F). Overexpression of α-CatR-ΔM1 in a wild-type background also resulted in a nearly two-fold increase in junctional Jub::GFP levels although Arm levels remained unchanged (S6C and S6D Fig). Consistent with this we found that the wing discs overexpressing α-CatR-ΔM1 in a wild-type background were significantly larger than wild-type control wing discs (S6B Fig).

Together, these findings indicate that the M region, and all three M domains are required to stabilize the CCC. However, loss of the M region does not deplete the CCC sufficiently to compromise epithelial integrity in the wing disc. In each case, including the α-CatR control, we found that Jub levels did not correspond to the observed reduction in Arm levels and in case of α-CatR and α-CatR-ΔM moderately exceed wild-type levels. Moreover, the striking increase in Jub enrichment at AJs with α-CatR-ΔM1 indicates that M1 strongly limits the junctional recruitment or stability of Jub. This explains why α-CatR-ΔM1 fails to rescue the tissue overgrowth resulting from α-Cat depletion (Fig 6), elicits tissue overgrowth when overexpressed (S6 Fig), and suggests that removing M1 decouples Jub recruitment to AJs from tissue tension. These conclusions are supported by the observed suppression of α-Cat-ΔM1 elicited overgrowth when Yki or Jub were reduced (Fig 6B and 6D). The fact that the loss of the entire M region does not adversely affect the regulation of tissue growth may be the result of compensatory activities of M1, which normally limits junctional localization of Jub, and M2 and M3, which normally appear to enhance junctional localization of Jub (Fig 7F). We conclude that the M region of α-Cat has complex activities that contribute to the regulation of AJ stability and the Jub recruitment-dependent regulation of tissue growth.

### Vinculin is not required for the α-CatR-ΔM1 elicited Jub hyper-recruitment or tissue overgrowth

The striking impact of deleting M1 on Jub junctional localization prompted us to ask whether Vinculin is involved as Vinculin is a well-established binding partner of M1 [6, 24, 25, 48]. Drosophila *Vinculin* (*Vinc)* null mutants are homozygous viable and fertile [49, 50]. We found Jub::GFP levels were not increased in *Vinc* mutant 3rd larval wing discs compared to controls (Fig 7G and 7H), and wing discs were not enlarged compared to wild-type (Fig 7I). These findings suggest that Vinc does not play an essential role in tissue growth of the wing disc epithelium and that the mechanism explaining how M1 limits Jub recruitment to AJs does not appear to involve its interaction with Vinc.

### Mechanosensing of the α-Cat actin-binding domain is required for normal growth regulation

We recently showed that the ABD of α-catenin proteins has mechanosensory properties that underpin the apparent catch-bond behavior of α-catenin ABD [7, 9]. The first α-helix of the α-catenin ABD, α1-helix, undergoes a conformational change under physiological tension, consequently revealing an enhanced actin-binding interface and a small β-sheet hairpin loop (βH) that facilitates ABD dimerization (see Fig 4A for the location of sequence elements). In vitro actin-binding assays suggest that a mutation (called H1) in the α1-helix that compromises its helical structure or deletion of the α1-helix enhance actin-binding and bundling activities, whereas deletion of βH (ΔβH) or a combination of H1 and ΔβH reduces actin-binding [9]. Thus, in contrast to removal of the whole ABD, which renders α-Cat non-functional [9, 43], H1 and ΔβH provide more subtle changes in ABD activity that either enhance or weaken the direct interaction of α-catenin with F-actin. The corresponding mutant fly isoforms (α-CatR-H1, α-CatR-ΔβH, α-CatR-H1-ΔβH), as well as isoforms that compromise the actin-binding interface (L798A+I805A+V809A = α-CatR-3A), or lack ABD (α-CatR-ΔABD) were all effectively recruited to AJs (S5 Fig) [9]. In contrast to α-CatR expressing animals, which rescue *α-Cat* mutants to adults, α-CatR-3A, α-CatR-ΔABD, and α-CatR-ΔβH did not rescue the embryonic lethal phenotype of *α-Cat* zygotic mutants. α-CatR-H1 and α-CatR-H1-ΔβH showed some rescue activity, with α-CatR-H1-ΔβH having a better rescue than α-CatR-H1. This is consistent with the notion that the H1 mutation, which enhances actin-binding, and the ΔβH mutation, which reduces actin binding, may compensate for each other to some degree [9].

**Fig 8:**
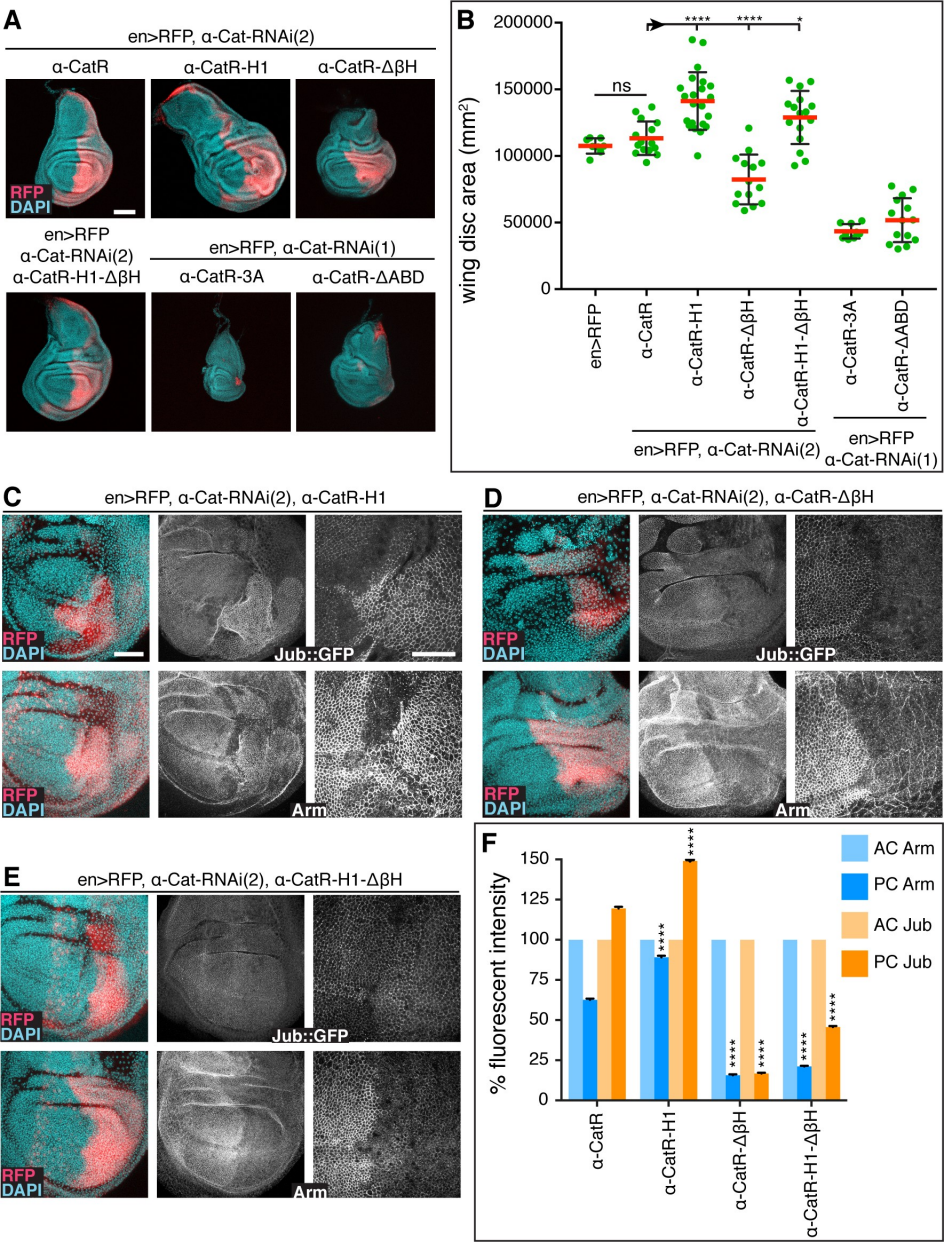
The mechanosensory α1-helix of the α-Cat ABD regulates tissue growth. (A, B) Sample discs (A) and quantification of disc area (B) of late 3^rd^ larval instar wing discs of the indicated genotypes. Note that UAS-α-CatR-H1 expression in *en-Gal4*, *UAS-α-Cat-RNAi(2)* discs causes hyperplastic overgrowth. α-CatR-3A and α-CatR-ΔABD expression enhanced the phenotype of *en>α-Cat-RNAi(1)* discs. 3^rd^ larval discs expressing α-CatR-3A in a *en>α-Cat-RNAi(2)* background did not develop. Two-tailed, unpaired t-test; ****(P≤0.0001), *(P = 0.0148), ns (P>0.05). Scale bars, 100 μm. (C-E) Late 3^rd^ larval instar discs expressing *en-Gal4*, *UAS-RFP*, *UAS-α-Cat-RNAi(2)* and either *UAS-α-CatR-H1* (C), *UAS-α-Cat-ΔβH* (D), or *UAS-α-CatR-H1-ΔβH* (E). Discs express Jub::GFP controlled by its endogenous promoter (upper panels) and are stained for Arm (lower panels). Nuclei are labeled with DAPI. Close-up images to the right show wing pouch area on both sides of the anterior-posterior compartment boundary. Scale bars, 50 μm and 25 μm. (F) Comparison of relative fluorescent intensities between AC and PC for Jub::GFP (N = 500–1000 cells from five wing discs) and Arm (N = 500–1000 cells from three to four wing discs). AC values were normalized to 100%. Mann Whitney test was used to determine statistical significance comparing experimental to control (α-CatR) discs. ****(P≤0.0001).

We could not recover 3^rd^ instar larvae with *en>α-Cat-RNAi(2) α-CatR-3A*. We therefore tested α-CatR-3A as well as α-CatR-ΔABD in a background expressing the weaker *α-Cat-RNAi(1)* line. *en>α-Cat-RNAi(1)* on its own caused hyperplastic overgrowth (Fig 1C and 1E). In contrast, in conjunction with α-CatR-3A or α-CatR-ΔABD we recovered discs that essentially lacked a PC (Fig 8A and 8B) similar to discs expressing *en>α-Cat-RNAi(2)*. These observations further emphasize that direct actin binding is critical for α-Cat function in vivo [9, 43], and suggest that α-CatR-3A and α-CatR-ΔABD act as dominant-negative isoforms of α-Cat, interfering with the function of the endogenous protein that is not fully removed by *α-Cat-RNAi(1)*.

Expression of α-CatR-H1, α-CatR-ΔβH, and α-CatR-H1-ΔβH together with *en>α-Cat-RNAi(2)* in the wing imaginal disc substantially improved disc morphology (Fig 8A and 8B) compared to *en>α-Cat-RNAi(2)* discs that had lost most of their PCs (Figs 1E and 6A). α-CatR-H1, α-CatR-ΔβH, and α-CatR-H1-ΔβH showed intriguing differences in their ability to rescue the depletion of the endogenous protein. *en>α-Cat-RNAi(2) α-CatR-ΔβH* discs showed normal epithelial organization of the PC but overall disc size remained smaller than of α-CatR controls. *en>α-Cat-RNAi(2) α-CatR-H1* discs were overgrown similar to *en>α-Cat-RNAi(1)* discs, and showed upregulation of the Yki transcriptional reporter *ex-lacZ* (Fig 8A and 8B; S7 Fig). These findings suggest that α-CatR-H1 supports normal epithelial polarity but fails in growth regulation. Finally, *en>α-Cat-RNAi(2) α-CatR-H1-ΔβH* discs showed an amount of tissue overgrowth intermediate between α-CatR-H1 and α-CatR (Fig 8A and 8B), suggesting that α-CatR-H1-ΔβH supports epithelial integrity and, to a larger extent than α-CatR-H1, the normal growth regulatory function of α-Cat.

In comparing junctional Jub::GFP and Arm levels in *en>α-Cat-RNAi(2)* discs expressing α-CatR-ΔβH or α-CatR-H1-ΔβH we found that Arm was reduced to 18% and 21% in PC compared to AC cells, respectively (Fig 8D–8F). This suggests that these mutant forms do not support normal stability of the CCC, and consequently cause significantly lower junctional Jub levels of 17% and 46% compared to controls. In contrast, α-CatR-H1 discs displayed higher than α-CatR control levels of Arm (88% versus 66%) and Jub (149% versus 119%) (Fig 8C and 8F). This is interesting as it suggests that the enhanced actin-binding activity observed for α-CatR-H1 in vitro [9] causes the formation of more stable AJs in vivo, which could result in the observed enrichment of junctional Jub. The elevated recruitment of Jub to AJs likely accounts for the observed tissue overgrowth of *en>α-Cat-RNAi(2) α-CatR-H1* discs (Fig 8A and 8B). Together, these results suggest that mechanosensing of the α-Cat ABD and the precise mechanism of the α-Cat F-actin association are important components of the AJ-dependent regulation of tissue growth.

### Loss of α-Cat mechanosensing does not change tissue tension

**Fig 9:**
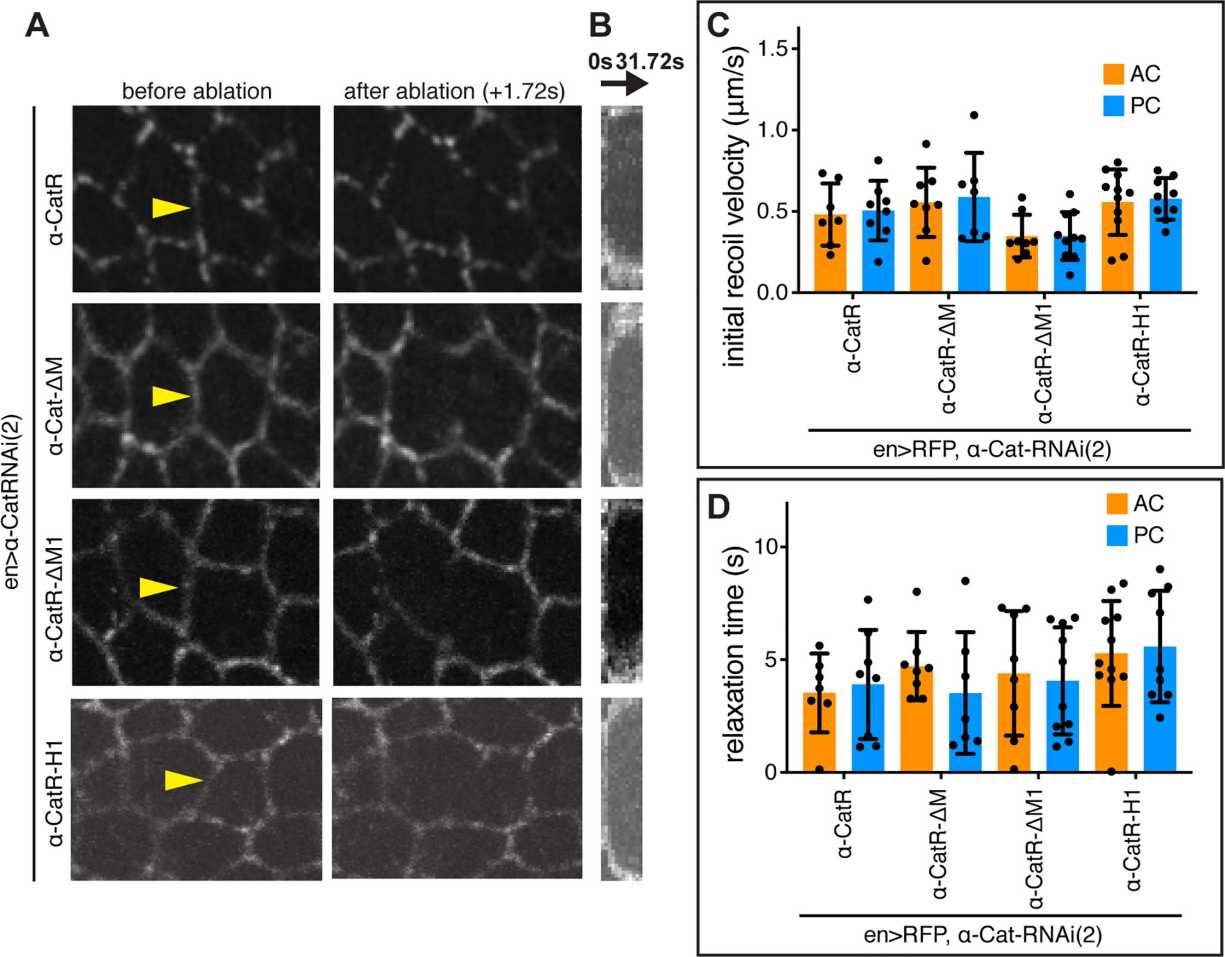
α-Cat mechanosensory mutants do not alter tissue tension. (A) Late 3rd larval instar wing imaginal discs expressing Jub::GFP, *en-Gal4*, *UAS-RFP*, *UAS-α-Cat-RNAi(2)*, and either *UAS-α-CatR*, *UAS-α-Cat-ΔM*, *UAS-α-CatR-ΔM1*, or *UAS-α-CatR-H1*. Junctions were cut with a laser pulse (see Materials and methods) at the sites indicated by the arrowheads. Initial recoil velocity (C) and relaxation time (D) was determined from movie sequences. (B) Kymographs demonstrating the displacement of adjacent cell vertices of the ablated cell edge shown in (A) which was used to determine the relaxation time (D). (C,D) Quantification of initial recoil velocities (C) and relaxation time (D) of cell edges after laser ablation in wing discs of indicated genotypes. 3–5 discs were used per genotype, and only data for which relaxation times could be calculated with confidence were included. No significant differences between anterior and posterior cell edges were observed for either dataset (Mann-Whitney test).

The tension generated by cytoskeletal forces recruits Jub to AJs [10, 51] and increases cell proliferation in the wing disc [27]. Therefore, one possible explanation for the excessive recruitment of Jub to AJs by some α-Cat mutant isoforms is that these α-Cat proteins do not have a direct effect on Jub localization but act indirectly through an increase in tissue tension. To address this question, we determined the initial recoil velocity and relaxation times after laser ablation of cell junctions. Experimental PC cells were compared to control AC cells in discs expressing α-CatR, α-CatR-ΔM, α-CatR-ΔM1, and α-CatR-H1 in an *en>α-Cat-RNAi(2)* Jub::GFP background. We found no significant differences in comparing the initial recoil velocities of AC control cells to PC experimental cells (Fig 9), suggesting that the impact of the tested α-Cat mutant isoforms on growth is not through a change in tissue tension. In addition, we used a Kelvin-Voigt model to quantify the viscosity-to-elasticity ratio at the site of ablation, by measuring the relaxation times of the displacements caused by laser severing [52, 53]. These tests did not reveal significant differences in relaxation times between any of the conditions, which suggests that the ratio of viscosity to elasticity is unaffected by these α-Cat mutant isoforms. Our observations suggest that the robust AJs observed in cells expressing α-CatR-H1 (Fig 8) do not significantly change tension or tissue viscosity. Furthermore, results from laser cut experiments further argue that Jub recruitment to AJs has been decoupled from tissue tension when M1 is removed, and instead Jub is constitutively recruited to the junction regardless of the degree of tension. These findings support the view that α-Cat acts as a mechanosensor that translates cytoskeletal tension into biochemical signals that limit tissue growth.

## Discussion

One key factor that determines the impact of α-Cat on tissue growth, leading either to overgrowth or tissue degeneration, is the amount of α-Cat at AJs. Analysis of phenotypic defects resulting from the differential reduction of gene function revealed that Drosophila α-Cat is a negative regulator of tissue growth. Moderate α-Cat overexpression or moderate depletion of α-Cat or DEcad caused Yki activation and overproliferation. Moreover, consistent with our findings, a previous analysis of strong loss-of-function conditions for α-Cat, DEcad, or Arm led to the conclusion that a loss of the CCC reduces Yki activity and causes JNK-meditated tissue degeneration [22]. This conclusion was at odds with some mammalian studies where the loss of E-cadherin or αE-catenin causes YAP or TAZ-dependent overgrowth in a number of different tissues and cell lines [for review see 14, 54]. Our data showing that α-Cat and DEcad limit Yki activity and tissue growth in Drosophila similar to mammalian tissues suggest a conserved functional relationship between AJs and the regulation of Yki/YAP/TAZ activity. Only when the Drosophila CCC is strongly depleted does a substantive activation of JNK signaling override Yki activation, causing tissue degeneration. This could potentially involve a direct inhibition of Yki by JNK [55].

**Fig 10:**
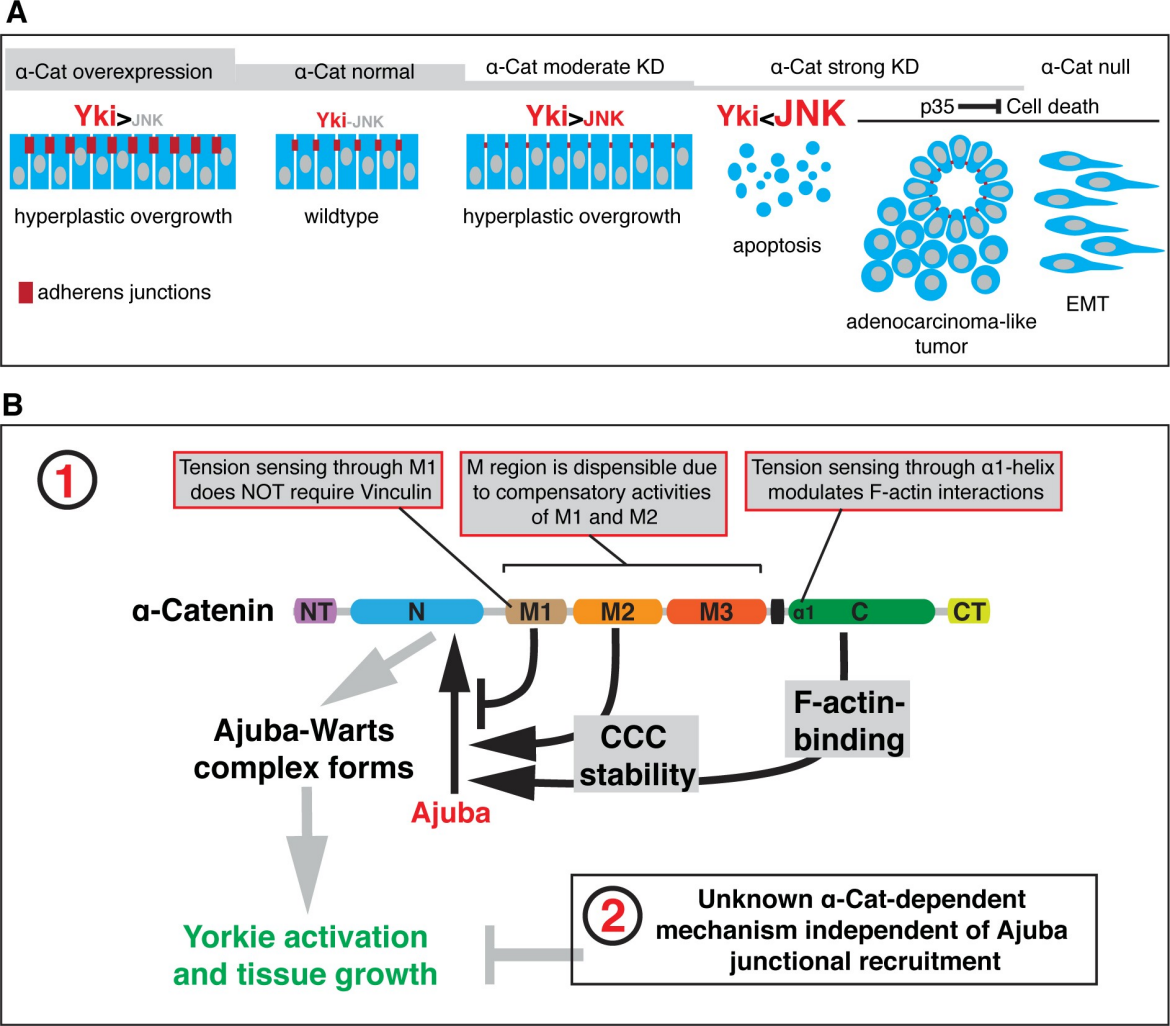
Model for α-Cat function in the regulation of tissue growth. (A) Illustration of the α-Cat phenotypic series and model for the differential activation of JNK and Yki signaling in response to changes in α-Cat levels (see text for further discussion). (B) Schematic model of α-Cat function in the regulation of tissue growth of the wing disc epithelium. α-Cat regulates tissue growth through Ajuba (Jub)-dependent (1) and Jub-independent (2) mechanisms. M1 limits Jub recruitment to the N domain of α-Cat by a mechanism that is not understood, but does not involved the main known binding partner of M1, Vinc. Loss of M1 compromised M region mechanosensing and led to a high-level constitutive recruitment of Jub that is independent of tissue tension. The M2 domain (and to a lesser extent M3) and the α-Cat ABD regulate stability of the CCC, with lower CCC levels at AJs reducing Jub recruitment. Loss of ABD mechanosensitivity by compromising α1-helix causes increased F-actin binding and stabilizes the CCC, leading to enhanced Jub recruitment and consequently tissue growth.

α-Cat loss-of-function conditions in conjunction with a block of cell death defined three distinct phenotypic classes that broadly align with the progression sequence of epithelial cancer from adenoma (epithelial overgrowth), to adenocarcinoma (overgrowth associated with a partial loss of epithelial integrity), to carcinoma (loss of epithelial integrity with cells showing protrusive activity) (Fig 10A). Similarly, in mammalian cancer, such as human colon cancer is the down-regulation of αE-catenin associated with an increased propensity for cells to become invasive [reviewed in 56]. On the other hand, loss of αE-catenin was reported to suppress colorectal adenomas induced by APC loss-of-function [57], an effect that could be mediated by the Rho-Rho kinase signalling-dependent cell death elicited by the loss of the CCC in an APC mutant background [35]. Also in Drosophila, we found that Rho1 is required for cell death resulting from the loss of α-Cat, likely mediated by the Rho1 signalling dependent activation of the JNK pathway [36, 37, 58].

Loss of α-Cat leads to a corresponding decline of DEcad and Arm at AJs. Quantification of CCC proteins in the late stage embryonic epidermis of zygotic *α-Cat* null mutants suggested that AJs can be retained and support normal tissue architecture when CCC levels are reduced to less than 10% of normal [30]. As heterozygous *α-Cat* animals are normal, we anticipate that reduction of α-Cat to somewhere between 50% and 10% of wild-type levels will cross a threshold that will increase Yki activity without activating JNK signaling sufficiently to cause cell death (Fig 10A). How strongly α-Cat and DEcad have to be reduced to cause an activation of Yki remains to be explored. For example, reducing α-Cat could compromise the interactions between the AJ protein Echinoid and Salvadore, a Hippo binding partner important for normal Hippo activity [59], or compromise the interactions between Crb and Ex that could deregulate Hippo signaling [60–62]. Loss of α-Cat could also affect actin polymerization as mature AJs suppress actin polymerization [30, 63, 64], whereas enhanced actin polymerization is a known activator of Yki [14, 65, 66]. Reducing α-Cat could therefore directly promote actin polymerization prior to an overt defect in cell adhesion, and consequently stimulate Yki activity and tissue growth. Finally, loss of α-Cat may affect Yki not through the Hippo pathway as was documented for αE-catenin in mammalian keratinocytes [18].

Mammalian studies raised the possibility that αE-catenin could act independently of AJs to regulate tissue growth [reviewed in 56, 67]. In contrast, several observations argue that Drosophila α-Cat acts as an AJ component to limit tumorigenesis: (i) The mechanosensory properties of α-Cat regulate the recruitment of Jub to AJs, which stimulates Yki activity and tissue growth and implies that α-Cat is suspended between the E-cadherin/β-catenin complex and actomyosin [40; this work]. In particular, α-Cat mutants that disrupt mechanosensing and cause overgrowth (α-CatR-ΔM1 and α-CatR-H1) are effectively recruited to AJs where they support normal epithelial organization, suggesting that a specific junctional defect disrupts growth regulation. (ii) A reduction of DEcad is also associated with hyperplastic tissue overgrowth similar to the partial loss of α-Cat. (iii) The expression of a DEcad::αCat fusion protein restores both the epithelial organization and normal growth of α-Cat compromised wing discs, and rescues *α-Cat* null mutant clones which normally fail to develop in wing discs [30]. Together, these data suggest that α-Cat acts as a component of the CCC at AJs to regulate tissue growth.

The mechanosensitive interactions between Jub and α-Cat are thought to transmit tissue tension into growth regulatory signals. Cytoskeletal tension enhances recruitment of Jub to junctional α-Cat where Jub forms a complex with Wts, preventing it from phosphorylating and hence deactivating Yki [10, 21, 68, 69]. Supporting this model, we observed Jub and Yki-dependent tissue overgrowth that correlates with an enhanced recruitment of Jub to AJs. In particular, our data suggest that the M1 domain acts as a gatekeeper for Jub recruitment (Fig 10B). In the absence of M1, junctional Jub levels become strikingly high, suggesting that M region mechanosensing has become ineffective in limiting Jub recruitment, causing Yki activation and overgrowth [see also 40]. Tension is thought to cause a conformational change in the M region and an unfurling of the M1 domain exposing a Vinc binding site [6, 8, 25, 70, 71]. However, Vinc is not required for limiting Jub recruitment and deletion of M1 causes a reduction of junctional Vinc but not a complete loss [40]. This leaves the mechanism of how M1 moderates Jub recruitment unresolved. A second unknown binding partner of M1 may be involved or an intramolecular interaction between M1 and the Jub binding site in the N domain [39, 40; this work] could control Jub binding.

This Jub recruitment-dependent model of how mechanotransduction by the α-Cat M region regulates tissue growth does not explain all our observations and therefore needs to be extended to incorporate additional mechanisms of how AJs can control tissue growth (Fig 10B). First, this model cannot explain the Yki-dependent overgrowth precipitated by low α-Cat levels, and corresponding low Jub levels at AJs. Second, we observed that two fusion proteins between DEcad and α-Cat, one containing both full-length proteins (DEcad::αCat) and one lacking the Jub binding domain in α-Cat (DEcadΔβ::αCatΔN), can both support normal growth in α-Cat KD tissue but only DEcad::αCat restored normal junctional Jub levels whereas DEcadΔβ::αCatΔN did not. Third, expression of α-CatR in α-Cat KD tissue restores α-Cat to approximately 63% of normal levels. However, Jub increases to 119% without a noticeable increase in tissue size. One possibility is that mechanical force distributed over fewer α-Cat molecules enhances the mechanosensory response of individual α-Cat molecules in a non-linear manner resulting in higher Jub recruitment. Evidence for such a mechanism was recently reported for the recruitment of Vinc to AJs in the Drosophila germband [72], and may be similar for Jub recruitment to AJ in that tissue [51]. Fourth, removing the entire M region results in an α-Cat protein that can support normal wing development, implying that M region mechanosensing is not an essential aspect of regulating tissue growth. In light of the results with α-CatR-ΔM1, this can only be explained by assuming that removing M2 and M3 in addition to M1 has a compensatory effect. Whereas α-CatR and α-CatR-ΔM expression in α-Cat depleted PC tissue enhances Jub recruitment above AC control levels, loss of M2 or M3 causes Jub levels to remain below AC levels suggesting that these two domains somehow normally support the ability of α-Cat to recruit Jub, possibly by supporting the stability of the CCC at AJs (Fig 10B). Collectively, our findings suggest that M region mechanosensing contributes to Jub recruitment and Hippo/Yki pathway regulation but that the actual mechanisms involved have considerable complexity and require further analysis to be resolved.

Increased levels of Jub were also observed in response to disrupting the mechanosensory properties of the α-Cat ABD. Disruption of the α1-helix of ABD did not only enhance F-actin binding in vitro [9] but also stabilized the CCC at AJs in the wing epithelium as suggested by our observations. A corresponding increase in Jub levels at AJs could account for the persistent overgrowth observed in α-Cat KD tissue expressing α1-helix compromised α-Cat (α-CatR-H1). Thus, the mechanosensory properties of the M region and the α-Cat ABD are both important for regulating Jub recruitment to AJs and growth regulation through the Hippo/Yki pathway (Fig 10B). As changes in tissue tension are thought to modulate the Hippo/Yki pathway through junctional recruitment of Jub [10, 27] we asked whether α-Cat mutants that compromise mechanosensing cause changes in tissue tension or viscoelasticity that could indirectly affect Jub recruitment to AJs. We did not observe such changes after laser ablation of cell-cell junction. This is consistent with the finding that replacing α-Cat with α-CatR-ΔM1 did not change junctional myosin II levels, the major tension generator in the wing disc epithelium [40]. Taken together, these data suggest a direct molecular role of α-Cat in Jub recruitment and strongly argue that α-Cat operates as a crucial mechanosensor to regulate tissue growth.

In summary, we conclude that α-Cat uses multiple mechanisms to act as an important regulator of tissue growth in the Drosophila wing disc epithelium (Fig 10). It is doing so at least in part by operating as a mechanotransducer, engaging both M region and ABD mechanosensing, to relay cytoskeletal tension into growth regulatory signals. One of these mechanisms involves the recruitment of Jub to the N domain of α-Cat that can be modulated by both mechanosensing mechanisms. However, α-Cat also engages mechanisms that are independent of the junctional recruitment of Jub to control tissue growth, which remain to be explored further.

## Material and methods

### Drosophila stocks

The fly stocks used were as follows: *Ubi*-α-*Cat*, *Tub-GAL80*, *FRT40A* [30], *hsFLP FRT40A;* α-*Cat*^*1*^, *da-GAL4*, *UAS-mCD8*::*GFP/TM6B* [30], *UAS-α-Cat-RNAi(1)/TM6B* (this work), *en-Gal4*, *UAS-RFP/CyO* (Bloomington Drosophila Stock Center (BDSC) 30557), *UAS-p35* [32], *puc*^*E697*^*-lacZ/TM6B* [34], *UAS-shg-RNAi* (Vienna Drosophila Resource Center (VDRC) 27081), *omb-Gal4*, *ex-lacZ/CyO* (BDSC 44248), *UAS-α-Cat-RNAi(2)* (TRiP HMS00317, Harvard Medical School), *UAS-jub-RNAi* (TRiP line HMS00714), *UAS-ex-RNAi* (TRiP line HMS00874), *UAS-crb-RNAi* (TRiP line JF02777), *UAS-ft-RNAi* (TRiP line HMS00932), *yki*^*B5*^*/CyO* [73], *Jub*::*GFP* [44], Rho1^72O^ (BDSC 7325), and *Vinc* [50]. Full genotypes for all figures can be found in S1 Table.

### Clonal analysis

*α-Cat*^*1*^ MARCM clones in imaginal discs were generated as described [30]. Briefly, to induce clones in wing discs, embryos were collected at 25°C for 24 hours, larvae were heat shocked twice at 37°C for 2 hours at 48 hrs after egg laying (AEL), and again at 72 hours AEL. Larvae were collected at 96 hrs AEL and dissected. Clones were induced in larvae of the following genotype:

- *hs-FLP*, *FRT40A/tub-Gal80*, *Ubi-α-Cat FRT40A; Act5c-Gal4 /da-Gal4*, *UAS-mCD8*::*GFP*, *αCat*^*1*^
- *hs-FLP*, *FRT40A/tub-Gal80*, *Ubi-α-Cat*, *FRT40A; Act5c-Gal4*, *α-Cat*^*1*^*/da-Gal4*, *UAS-mCD8*::*GFP*, *αCat*^*1*^ and
- *hs-FLP*, *FRT40A/tub-Gal80*, *Ubi-α-Cat FRT40A; Act5c-Gal4*, *UAS-p35*, *α-Cat*^*1*^*/da-Gal4*, *UAS-mCD8*::*GFP*, *α-Cat*^*1*^.

### Generation of *Drosophila* transgenes

To generate the *UASp-α-Cat* constructs, full-length α-Cat cDNA [74] (2751 nucleotides) was cloned into Gateway pENTR/D-TOPO entry vector (K240020, Thermo Fischer Scientific) digested with *Not*I and *Asc*I, using 3-part Gibson assembly reaction (New England Biolabs). α*Cat* cDNAs carrying various mutations (S2 Table) were cloned using 2-part or 3-part Gibson assembly reactions with *α-Cat* in pENTR/D-TOPO as the backbone digested with restriction enzymes. DNA fragments carrying the mutations were synthesized by Thermo Fischer. The Gateway LR Clonase Enzyme mix was used to clone all entry vector constructs into *pPWH-attB* (*pUASP-*Gateway Cassette with C-terminal 3xHA tag, #1102, Drosophila Genomics Resource Center modified by insertion of an *attB* recombination site [75] at a *Nsi*I restriction site. Transgenic animals were produced by Best Gene Inc., by using flies carrying the *attP2* recombination site.

*UAS-α-Cat-RNAi(1)* construct was designed by selecting a 21-nt target sequence 5’-GGTTAAAGAATTTATGTTAAA-3’ in the *α-Cat* transcript based on the algorithm as described [76]. The 71 basepair oligonucleotide sequence 5’-ctagcagtGGTTAAAGAATTTATGTTAAAtagttatattcaagcataTTTAACATAAATTCTTTAACCgcg-3’ with overhangs for the restriction enzymes *Nhe*1 and *Eco*R1 was synthesized by Thermo Fischer. The annealed oligonucleotides were cloned into the VALIUM20 vector (TRiP functional genomics resources, Harvard Medical School). To render α-CatR and derivative constructs resistant to the shRNAs *α-Cat-RNAi(1) and α-Cat-RNAi(2)* following modifications were made in the DNA sequence: the 5’-ATGTTAAAA-3’ sequence at the start of the *α-Cat* coding region, which is targeted by *UAS-α-Cat-RNAi(1)*, was changed to 5’-ATGCTGAAG-3’, and 5’-GCAGCATCGATATTGACTGTT-3’, which encodes sequences in the M2 domain and is targeted by *UAS-α-Cat-RNAi(2)* [TRiP line HMS00317] was modified to 5’-GCCGCCTCCATCCTGACCGTG-3’. The DEcadΔβ::αCat fusion protein was engineered as described previously [30]. To remove the Arm binding domain in DEcadΔβ amino acids 1445-AYEGDGNSDGSLSSLASCTDD-1466 were deleted from DEcad.

### Immunohistochemistry

Crosses using *α-CatRNAi(2)* were grown at 25°C. For *α-CatRNAi(1)*, embryos were collected for 24 hrs at 25°C and kept at 25°C until 48 hrs after egg laying (AEL), when they were moved to 29°C to enhance RNAi KD. Wing imaginal discs from late third instar larvae were dissected in cold Phosphate buffer saline (PBS), and fixed for 20 min in 4% paraformaldehyde in PBS at room temperature, then washed 3 times in 0.1% PBS-Triton X-100 followed by 30 minutes of incubation in 0.3% PBS-Triton X-100. Tissue was incubated with 5% goat serum in 0.1% PBS-Triton X-100 and then processed for antibody staining. Primary antibodies used were: guinea pig polyclonal antibody (pAb) anti-α-Catenin (p121; 1:1000, [30]), rat monoclonal antibody (mAb) anti-HA (3F10, 1:500, Sigma), mouse mAb anti-Arm (N2-7A1, 1:50, Developmental Studies Hybridoma Bank [DSHB]), mouse mAb anti-β-Galactosidase antibody (Z378A, 1:500, Promega), pAb rabbit anti-active JNK (V7931, 1:100, Promega) and pAb rat anti-Crb antibody (F3, 1:500, [77]). Fluorescent secondary antibodies were used at a dilution of 1:400 (Jackson Immuno Research Laboratories and Thermo Fisher). Larval tissues were further incubated with DAPI (1:1000, Molecular Probes) or Acti-stain Phalloidin (PHDG1-A, 1:50, Cytoskeleton Inc.), followed by washes in 0.1% PBS-Triton X-100. Tissues were incubated in Vectashield antifade mounting medium (H-1000, Vector Laboratories) overnight. Wing discs were mounted apical side up in Vectashield, with one layer of Scotch double-sided tape on both short edges of the coverslips (VWR 22x40 mm, No. 1.5) as spacers to prevent excessive compression of the discs as described [78]. For experiments in Fig 8G and 8H, male larvae of the genotypes, *en-Gal4 UAS-RFP/jub*::*GFP* and *Vinc; jub*::*GFP/+* were fixed and processed together in the same Eppendorf tube and mounted on the same slide.

### RNAi KD, overexpression, and genetic interaction experiments in the adult wing

All adult wing crosses were set up using the *omb-Gal4* driver. Embryos were collected for 24 hrs at 25°C, and kept at 25°C until eclosure of adult flies. Female flies were collected for 48 hrs after eclosure. Wings were then removed using forceps, washed in 0.1% PBS-Triton, and mounted in 50% Glycerol.

### Image acquisition, processing and quantification of imaginal discs and adult wings

Confocal images of wing discs were acquired on a Leica TCS SP8 scanning confocal microscope using HC PL APO CS2 20X/0.75 or HC PL APO CS2 40X/1.30 oil immersion objectives. Z-stacks were acquired to capture the full disc, and subsequent processing and maximal projections were made using the “Z-stack” function of Fiji (ImageJ). Total disc sizes were quantified by converting fluorescence to binary images using the Fiji threshold tool. Size measurements were made from outlines of total wing disc. Adult wing images were acquired on a Zeiss Axiophot microscope using the bright field mode, with a 5x lens (NA 0.15). Subsequent processing and wing size quantification was performed using the “measure” function of Fiji. Images were assembled in Adobe Photoshop and Adobe Illustrator. All graphs and statistical analyses were generated using Prism v7 (Graph Pad) software. We used unpaired two-tailed *t* tests to determine p-values when samples passed the normality test, and we used nonparametric two-sample Mann-Whitney test otherwise.

### Arm and Jub protein quantification

We used Scientific Image Segmentation and Analysis (SIESTA; [79, 80] to automatically identify cell outlines on confocal stacks of wing discs to determine Arm and Jub levels. 1-pixel-wide lines (60 nm/pixel) were selected to obtain the mean pixel intensity per cell. Using SIESTA, ~200 cells were selected above and below the dorso-ventral compartment boundary in both the anterior and posterior compartments. To eliminate background from measurements of protein levels, we subtracted the values of protein level measurements for the center of the cell from protein level measurements for the cell perimeter. In case of *UAS-α-Cat-ΔM2*, *UAS-α-CatR-ΔβH* and *UAS-α-CatR-H1-ΔβH*, anti-HA staining was used as a reference to identify cell outlines in the PC. For every larval wing disc, average protein measurements for the AC thus calculated were used to normalize the protein measurements per cell selected from the PC. Average protein measurements were then plotted as a percentage. Statistical significance was calculated using nonparametric two-sample Mann-Whitney test.

### Live imaging of wing discs and laser ablation

Wing discs from wandering 3rd instar larvae were dissected and mounted in a solution containing 2% heat inactivated FBS, 10 μg/ml insulin, and 1x Penicillin-Streptomyocin in Schneider’s medium. The discs were mounted between a cover glass and an oxygen permeable membrane. Imaging and laser ablation were done on a Revolution XD spinning disk confocal (Andor) with a 100× oil-immersion lens (NA 1.40; Olympus), iXon Ultra 897 camera (Andor) and Metamorph (Molecular Devices) image acquisition software. We collected 16-bit Z-stacks of 7 slices each, at 0.2 μm per step, taken every 3 seconds for 2.5 minutes. Cuts were done in cells within the pouch located several cell diameters away from the AP and DV boundaries. Cells roughly symmetrical in shape and without obvious polarization were chosen. We avoided junctions of disc pouch cells located immediately below junctions or nuclei of peripodial cells. A pulsed Micropoint N2 laser (Andor) tuned to 365 nm was used to apply ten laser pulses to a spot in the center of a single cell junction, with an image taken immediately before and after ablation (with 1.72s between images). Over the course of the movie, the positions of tricellular vertices which connected the ablated edge were manually tracked using SIESTA in order to determine initial recoil velocity and relaxation times. Positions were processed by MATLAB scripts based on a Kelvin-Voigt mechanical circuit, [53], whereby an edge is assumed to behave as a viscoelastic material, modelled as a spring and a dashpot configured in parallel. Statistical significance was accessed using nonparametric two-sample Mann-Whitney test.

### Whole-animal rescue experiments

Whole animal rescue experiments for *α-CatR*, *α-CatR-ΔM*, *DEcadΔβ*::*aCatΔN* were carried out as described [43].

## Supporting information

S1 Fig Expression of *α-Cat-RNAi(2)* leads to a stronger knockdown (KD) of α-Cat than *α-Cat-RNAi(1)*. (A,B) Cell clones in the larval wing disc generated using Actin-Gal4 flip out cassette and positively labelled with GFP expressing *α-Cat-RNAi(1)* (A) or *α-Cat-RNAi(2)* (B). Discs are labeled for DAPI and α-Cat. Note the low levels of α-Cat retained in *α-Cat-RNAi(1)* clones whereas α-Cat is undetectable in *α-Cat-RNAi(2)* clones. Also note that *α-Cat-RNAi(1)* clones remain fully integrated in the epithelium whereas *α-Cat-RNAi(2)* clones round up suggesting a more pronounced difference in cell adhesion between *α-Cat-RNAi(2)* cells and neighboring wild-type cells than between *α-Cat-RNAi(1)* cells and wild-type cells.
(TIF)

S2 Fig Whole animal rescue of α-CatR-ΔM and DEcad α-Cat fusion proteins. Whole animal survival plot showing average and total range of rescue activity of α-CatR, α-CatR-ΔM and DEcadΔβ::αCatΔN when expressed in *α-Cat* zygotic mutant embryos. Data are presented as mean±s.d. Two-tailed, unpaired t-test; ****(P≤0.0001). A score of 0 was given to *α*-*Cat*^1^ zygotic null mutant embryos which frequently displayed defects in head morphogenesis (‘head open’ phenotype). Embryos that displayed an enhancement of the *α*-*Cat*^1^ phenotype were given the following scores: (−2) embryonic lethal with both head open and a dorsal open phenotype indicating a failure of dorsal closure; (−1) embryonic lethal with both the head open defect and a hole in the dorsal epidermis indicating incomplete closure. For the rest of the animals that displayed rescue of the *α*-*Cat*^1^ phenotype following scoring criteria were used: (1) embryonic lethal with weak head defects (‘abnormal head’); (2) embryonic lethal with normal head; (3) lethal at first larval instar; (4) lethal at second larval instar; (5) lethal at third larval instar; (6) early pupa lethal; (7) late pupa lethal; (8) adult.
(TIF)

S3 Fig Overexpression of α-Cat causes overgrowth in a sensitized genetic background. (A,B) Depletion of Crb (A) or Ex (B) does not cause a significant enlargement of larval wing disc. However, overexpression of α-Cat in conjunction with a KD of Crb (A) and Ex (B) show synergistic overgrowth phenotypes (quantification shown in Fig 5D). Scale bars, 100 μm.
(TIF)

S4 Fig Levels of α-Cat are reduced compared to endogenous protein in α-Cat mutant cells expressing α-CatR. (A,B) Late 3^rd^ larval instar wing discs of indicated genotypes labeled with DAPI and posterior compartment (PC) marked by RFP. Close-up images to the right show wing pouch area on both sides of the anterior-posterior compartment boundary. α-Cat was depleted in PC with *α-Cat-RNAi(2)*. (C) Comparison of relative fluorescent intensities between anterior compartment (AC) and PC for α-Cat. AC values were normalized to 100%. N = 300–400 cells from two wing discs. Mann-Whitney test; ****(P≤0.0001). (D) Western blot analysis of protein levels of α-CatR and α-Cat using anti-HA antibody. β-tubulin is used as the loading control.
(TIF)

S5 Fig Expression of α-Cat constructs in wing imaginal discs. Late 3^rd^ larval instar wing discs of indicated genotypes were labeled with HA to detect the transgenic α-Cat protein and Arm. Data show that transgenic α-Cat proteins are effectively recruited to AJs.
(TIF)

S6 Fig Overexpression of α-CatR-ΔM1 reveals that the M1 domain is important for limiting tissue growth. (A) Adult wing area of flies overexpressing the indicated constructs with *omb-Gal4*. Two-tailed, unpaired t-test; ****(P≤0.0001). (B) Sample discs and quantification of late 3^rd^ larval instar wing discs of indicated genotypes labeled with DAPI and PC marked by RFP. Overexpression of α-Cat-ΔM1 results in hyperplastic overgrowth. Two-tailed, unpaired t-test; **(P≤0.01). Scale bars, 100 μm. (C) Discs expressing Jub::GFP controlled by its endogenous promoter (upper panels) and stained for Arm (lower panels). Nuclei are labeled with DAPI. Scale bars, 25 μm. (D) Comparison of relative fluorescent intensities between anterior compartment (AC) and posterior compartment (PC) for Jub::GFP (N = 500–600 cells from three wing discs) and Arm (N = 200–300 cells from two wing discs). AC values were normalized to 100%. Mann Whitney test was used to determine statistical significance between α-CatR-ΔM1 and control (α-CatR) discs. ****(P≤0.0001).
(TIF)

S7 Fig Wing discs expressing α-CatR-H1 in α-Cat mutant cells activate Yki. Late 3^rd^ larval instar wing discs of indicated genotypes labeled with DAPI and posterior compartment marked by RFP. Expression of α-Cat-H1 in an α*-Cat-RNAi(2)* background causes tissue overgrowth and elevated expression of *ex-lacZ*. Scale bars, 100 μm.
(TIF)

S1 Table List of genotypes for all figures. (PDF)

S2 Table α-Cat constructs used in this study. (PDF)
