# Supplementary figures and images for: Role of α-Catenin and its mechanosensing properties in regulating Hippo/YAP-dependent tissue growth
Source: PLoS Genet. 2019 Nov 7;15(11):e1008454. doi: 10.1371/journal.pgen.1008454 (PMC6863567; doi:10.1371/journal.pgen.1008454)

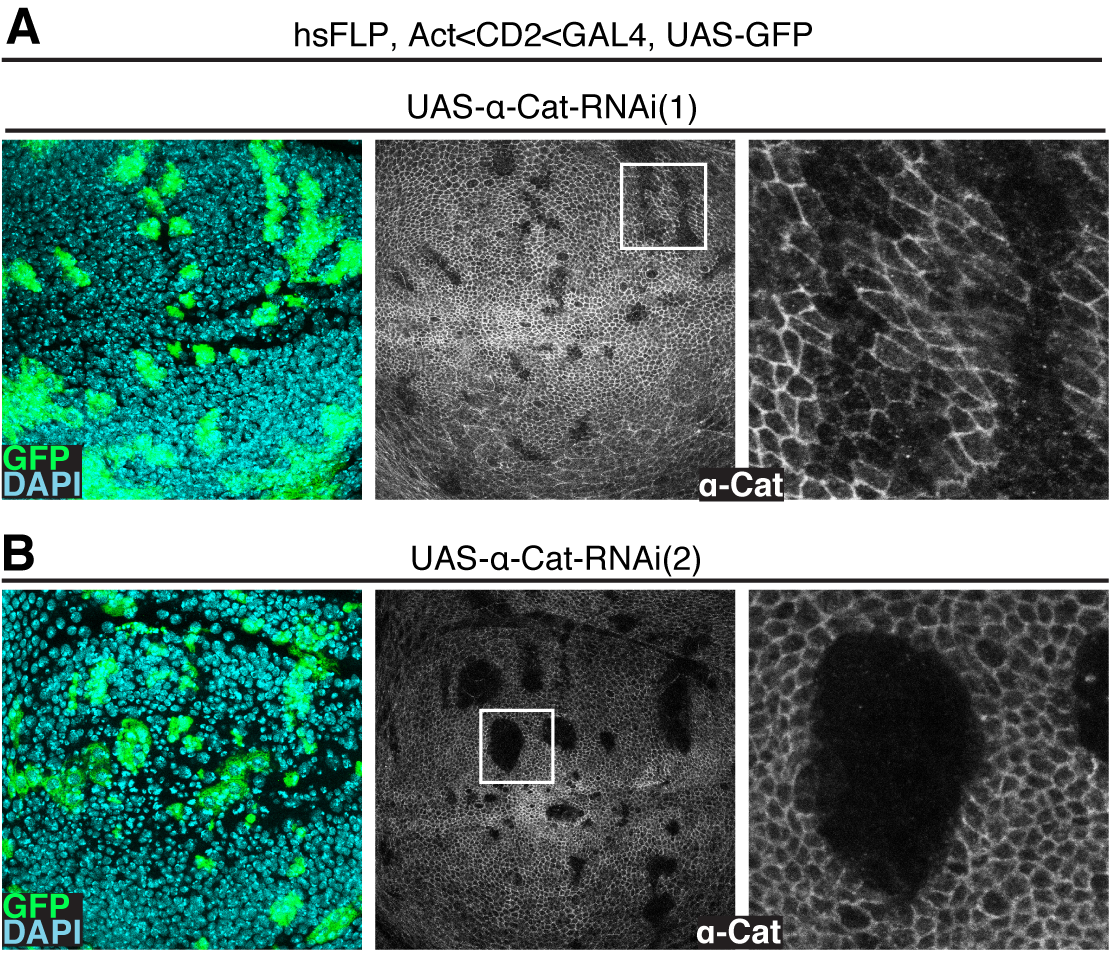

Supplement: S1 Fig — (A,B) Cell clones in the larval wing disc generated using Actin-Gal4 flip out cassette and positively labelled with GFP expressing α-Cat-RNAi(1) (A) or α-Cat-RNAi(2) (B). Discs are labeled for DAPI and α-Cat. Note the low levels of α-Cat retained in α-Cat-RNAi(1) clones whereas α-Cat is undetectable in α-Cat-RNAi(2) clones. Also note that α-Cat-RNAi(1) clones remain fully integrated in the epithelium whereas α-Cat-RNAi(2) clones round up suggesting a more pronounced difference in cell adhesion between α-Cat-RNAi(2) cells and neighboring wild-type cells than between α-Cat-RNAi(1) cells and wild-type cells. (TIF) [file pgen.1008454.s001.tif]

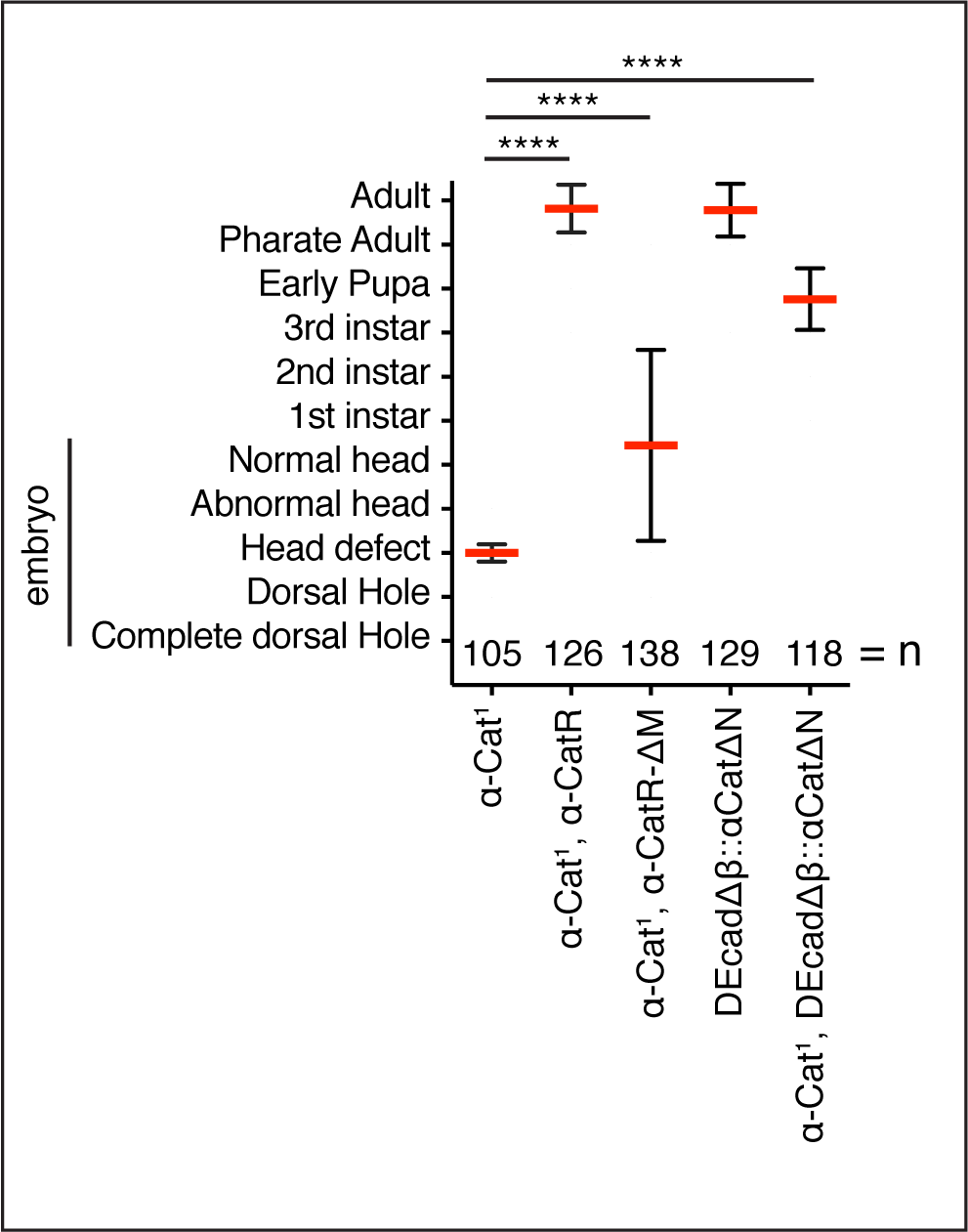

Supplement: S2 Fig — Whole animal survival plot showing average and total range of rescue activity of α-CatR, α-CatR-ΔM and DEcadΔβ::αCatΔN when expressed in α-Cat zygotic mutant embryos. Data are presented as mean±s.d. Two-tailed, unpaired t-test; ****(P≤0.0001). A score of 0 was given to α-Cat1 zygotic null mutant embryos which frequently displayed defects in head morphogenesis (‘head open’ phenotype). Embryos that displayed an enhancement of the α-Cat1 phenotype were given the following scores: (−2) embryonic lethal with both head open and a dorsal open phenotype indicating a failure of dorsal closure; (−1) embryonic lethal with both the head open defect and a hole in the dorsal epidermis indicating incomplete closure. For the rest of the animals that displayed rescue of the α-Cat1 phenotype following scoring criteria were used: (1) embryonic lethal with weak head defects (‘abnormal head’); (2) embryonic lethal with normal head; (3) lethal at first larval instar; (4) lethal at second larval instar; (5) lethal at third larval instar; (6) early pupa lethal; (7) late pupa lethal; (8) adult. (TIF) [file pgen.1008454.s002.tif]

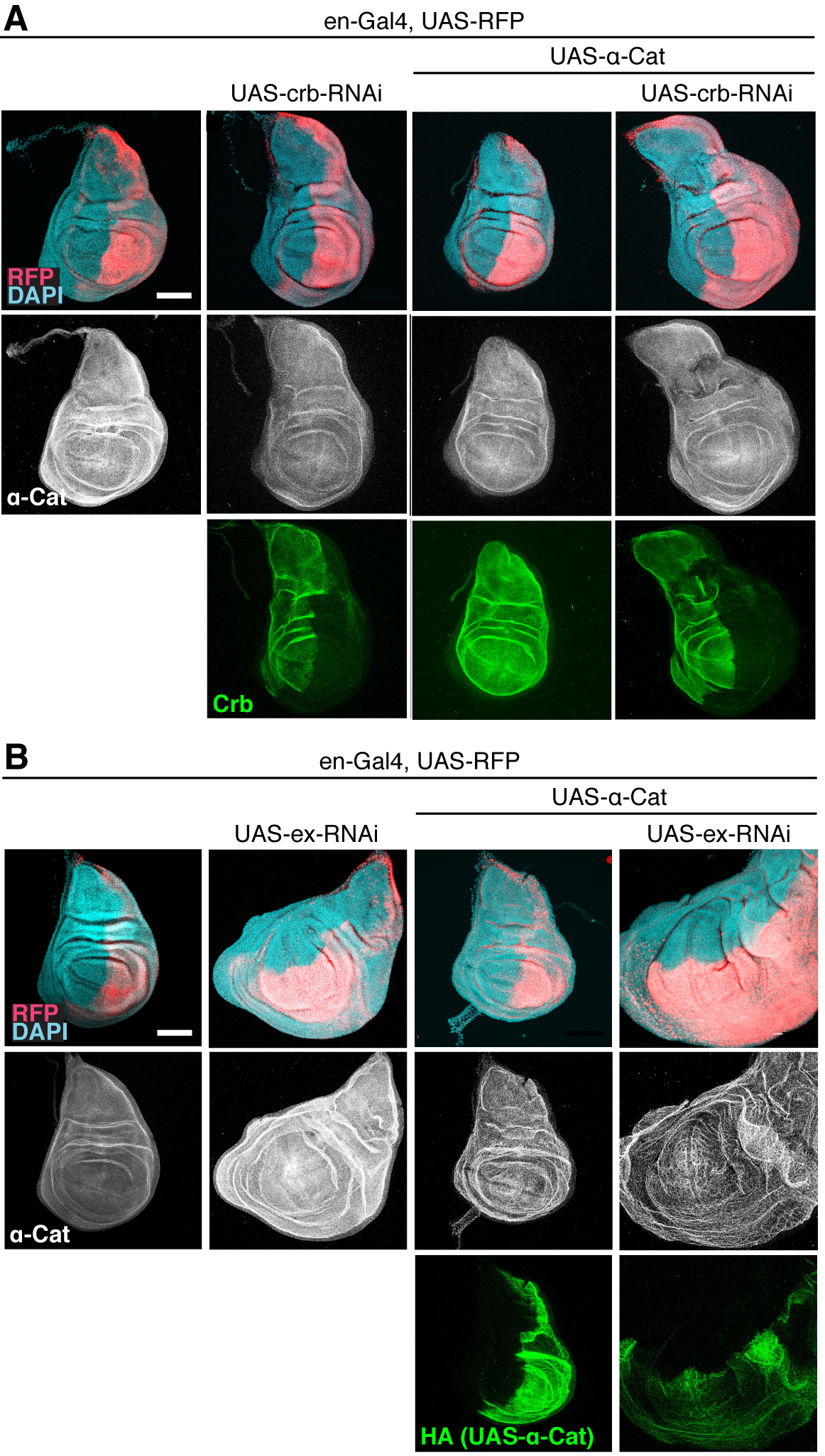

Supplement: S3 Fig — (A,B) Depletion of Crb (A) or Ex (B) does not cause a significant enlargement of larval wing disc. However, overexpression of α-Cat in conjunction with a KD of Crb (A) and Ex (B) show synergistic overgrowth phenotypes (quantification shown in Fig 5D). Scale bars, 100 μm. (TIF) [file pgen.1008454.s003.tif]

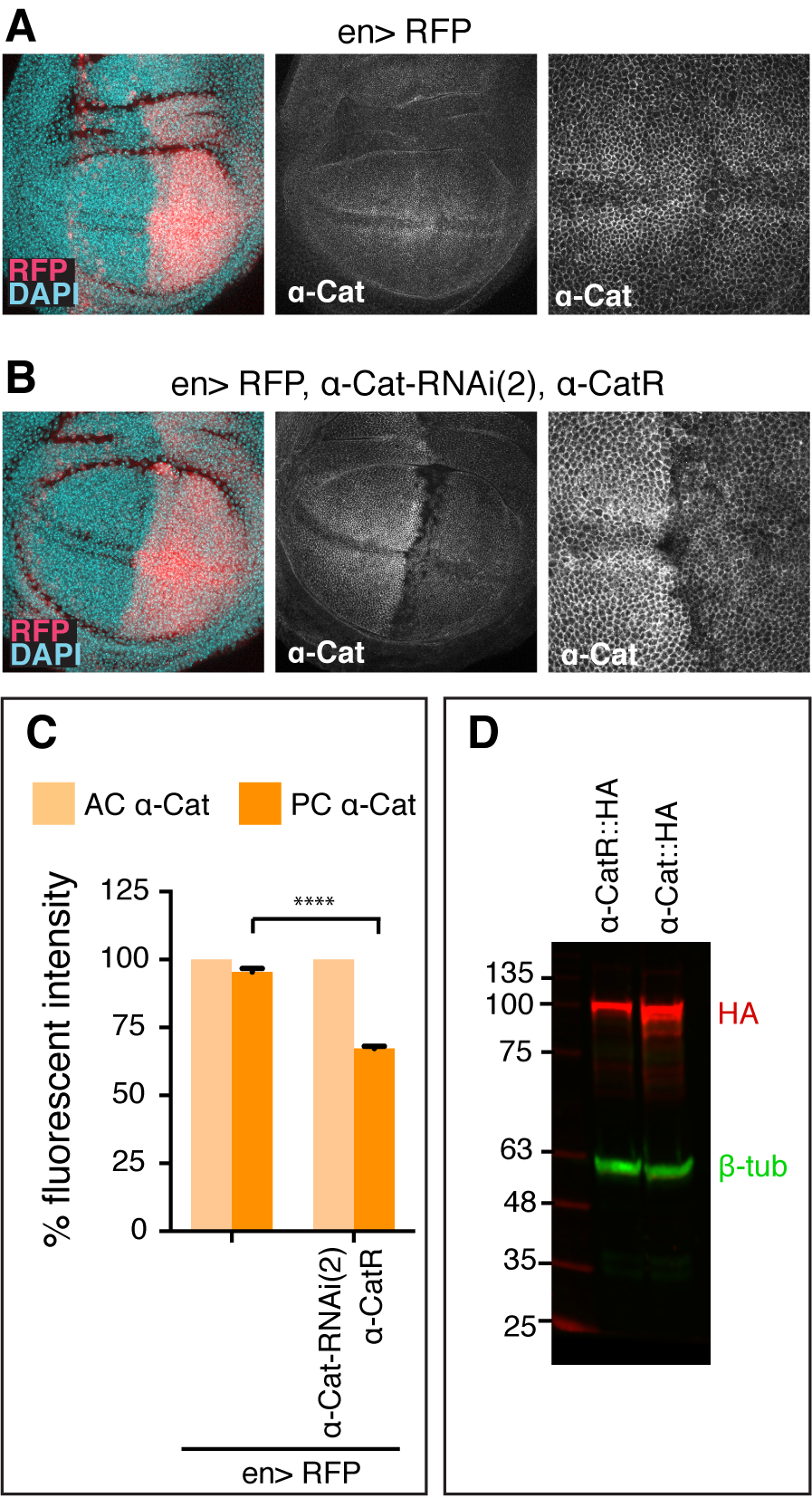

Supplement: S4 Fig — (A,B) Late 3rd larval instar wing discs of indicated genotypes labeled with DAPI and posterior compartment (PC) marked by RFP. Close-up images to the right show wing pouch area on both sides of the anterior-posterior compartment boundary. α-Cat was depleted in PC with α-Cat-RNAi(2). (C) Comparison of relative fluorescent intensities between anterior compartment (AC) and PC for α-Cat. AC values were normalized to 100%. N = 300–400 cells from two wing discs. Mann-Whitney test; ****(P≤0.0001). (D) Western blot analysis of protein levels of α-CatR and α-Cat using anti-HA antibody. β-tubulin is used as the loading control. (TIF) [file pgen.1008454.s004.tif]

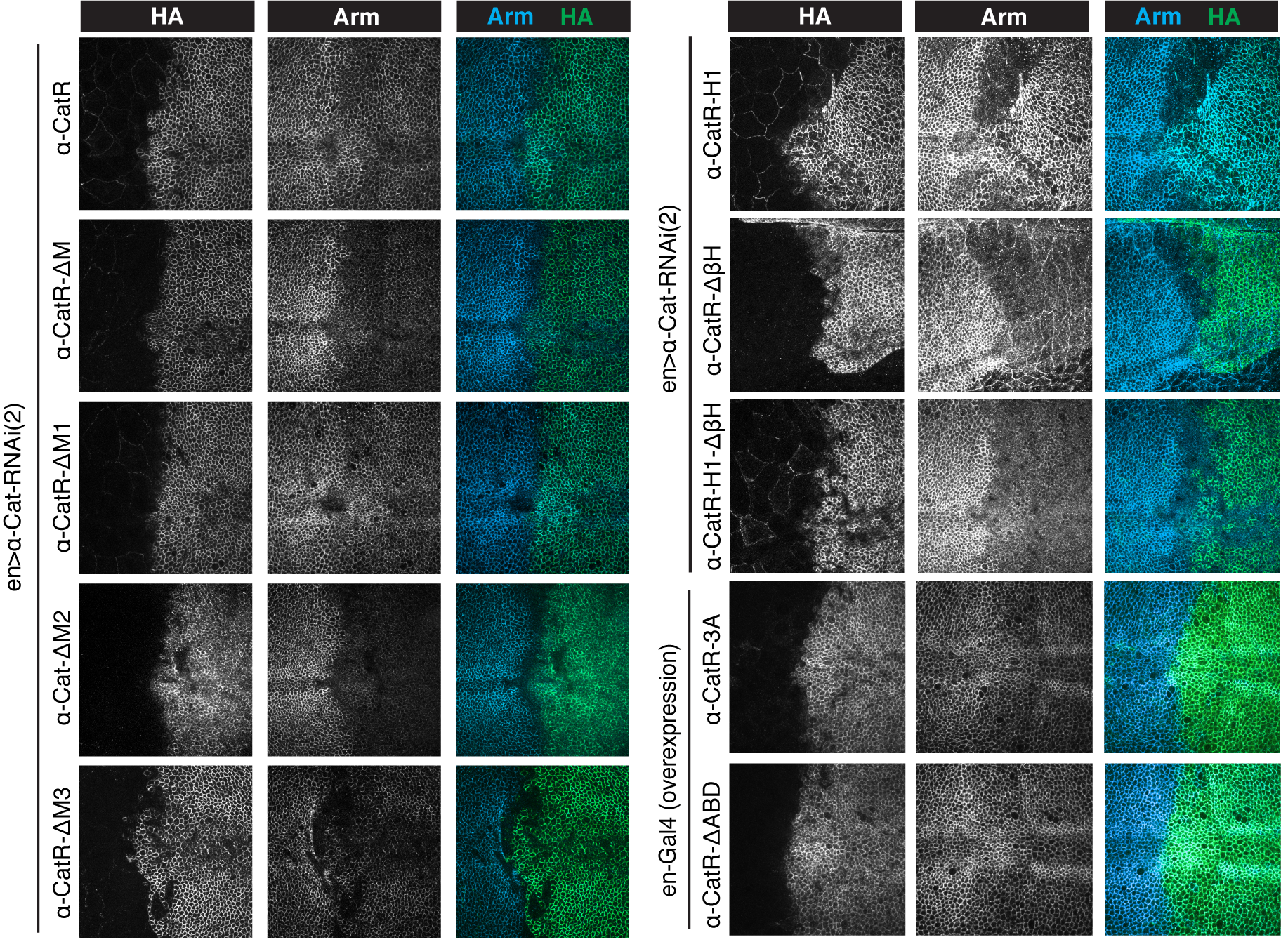

Supplement: S5 Fig — Late 3rd larval instar wing discs of indicated genotypes were labeled with HA to detect the transgenic α-Cat protein and Arm. Data show that transgenic α-Cat proteins are effectively recruited to AJs. (TIF) [file pgen.1008454.s005.tif]

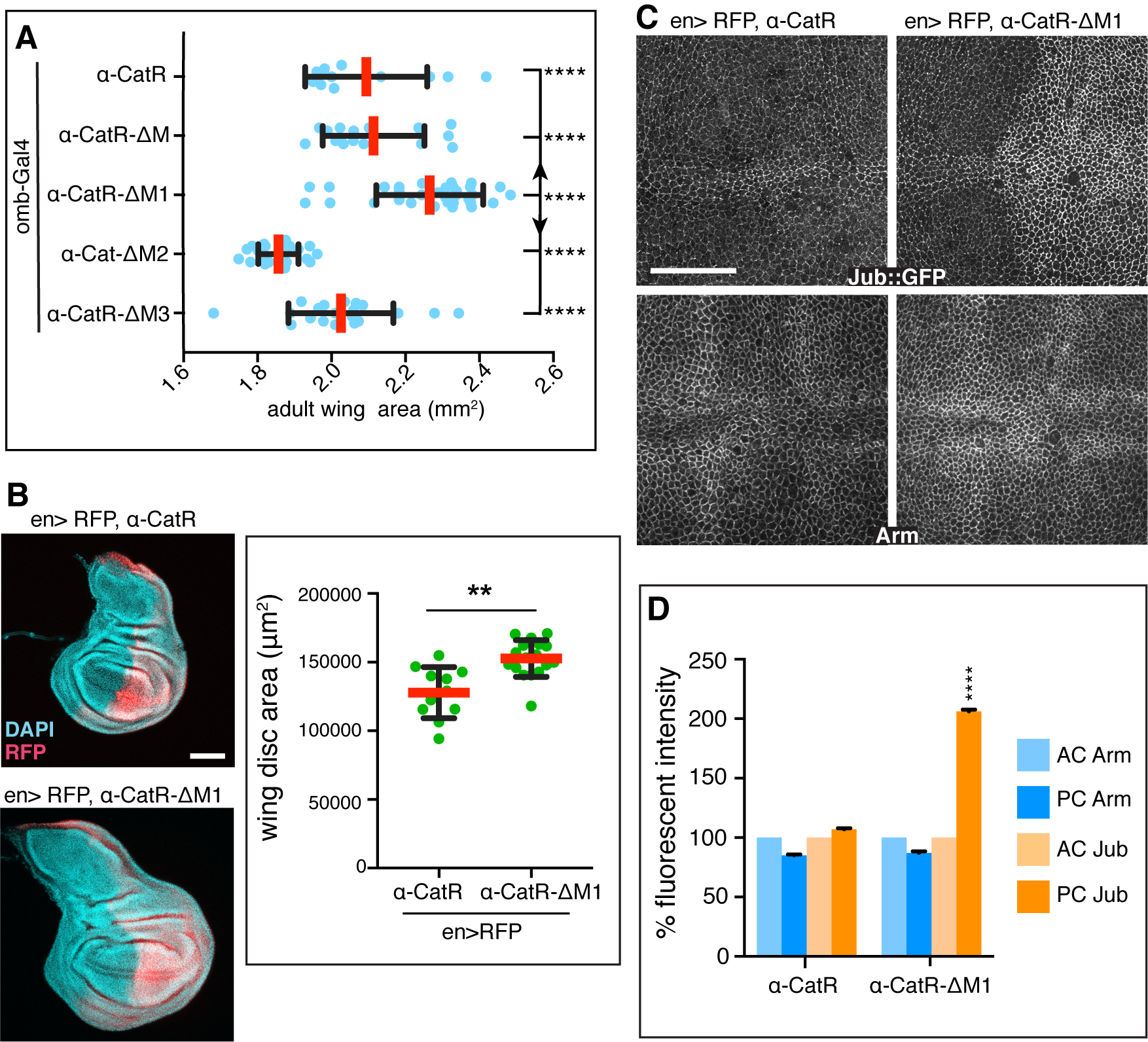

Supplement: S6 Fig — (A) Adult wing area of flies overexpressing the indicated constructs with omb-Gal4. Two-tailed, unpaired t-test; ****(P≤0.0001). (B) Sample discs and quantification of late 3rd larval instar wing discs of indicated genotypes labeled with DAPI and PC marked by RFP. Overexpression of α-Cat-ΔM1 results in hyperplastic overgrowth. Two-tailed, unpaired t-test; **(P≤0.01). Scale bars, 100 μm. (C) Discs expressing Jub::GFP controlled by its endogenous promoter (upper panels) and stained for Arm (lower panels). Nuclei are labeled with DAPI. Scale bars, 25 μm. (D) Comparison of relative fluorescent intensities between anterior compartment (AC) and posterior compartment (PC) for Jub::GFP (N = 500–600 cells from three wing discs) and Arm (N = 200–300 cells from two wing discs). AC values were normalized to 100%. Mann Whitney test was used to determine statistical significance between α-CatR-ΔM1 and control (α-CatR) discs. ****(P≤0.0001). (TIF) [file pgen.1008454.s006.tif]

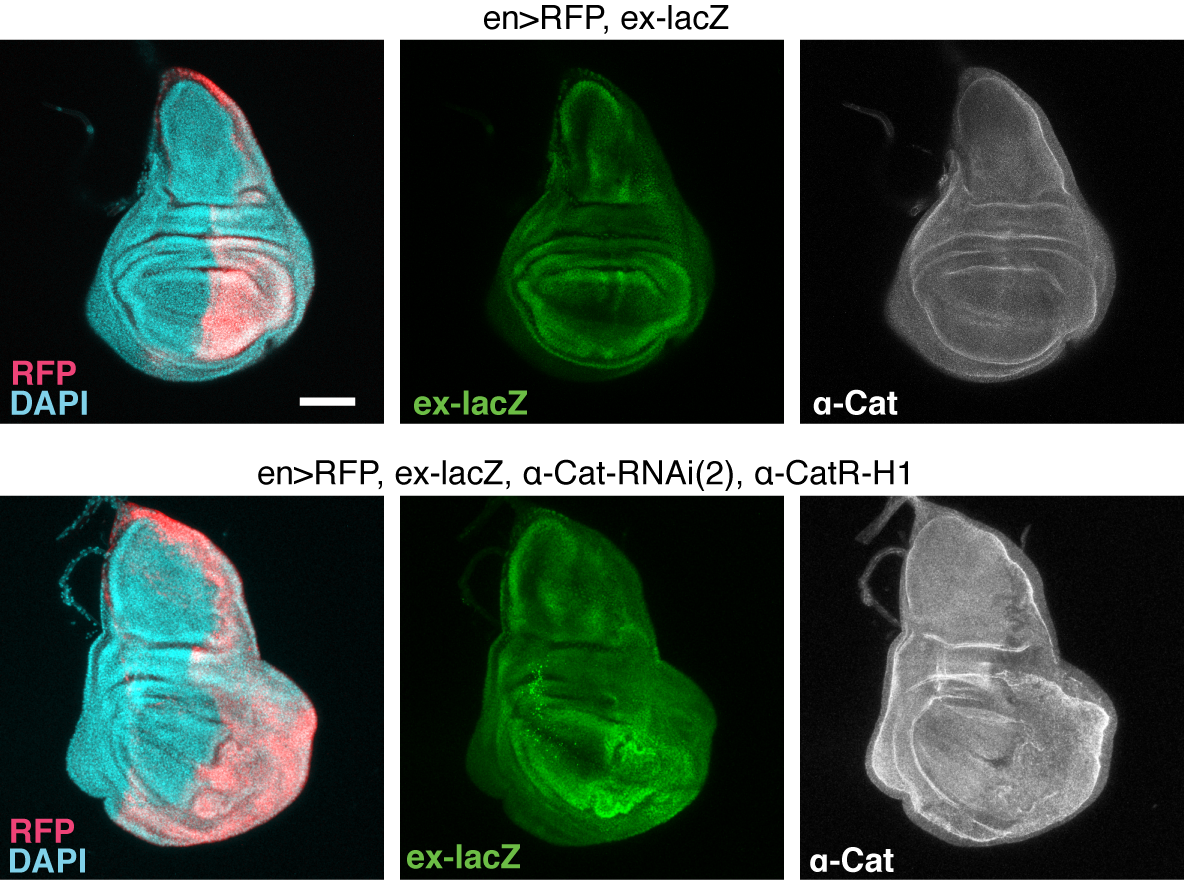

Supplement: S7 Fig — Late 3rd larval instar wing discs of indicated genotypes labeled with DAPI and posterior compartment marked by RFP. Expression of α-Cat-H1 in an α-Cat-RNAi(2) background causes tissue overgrowth and elevated expression of ex-lacZ. Scale bars, 100 μm. (TIF) [file pgen.1008454.s007.tif]
