# Supplementary material for: Role of α-Catenin and its mechanosensing properties in regulating Hippo/YAP-dependent tissue growth
Source: PLoS Genet. 2019 Nov 7;15(11):e1008454. doi: 10.1371/journal.pgen.1008454 (PMC6863567; doi:10.1371/journal.pgen.1008454)
Supplement: S1 Table — (PDF) [file pgen.1008454.s008.pdf]

S1 Table. List of genotypes

Fig 1

B

Gal80, ubi-αCat, FRT40A/hsFLP ; Act-Gal4, α-Cat^{1}/da-Gal4,UAS-GFP, α-Cat^{1}

Gal80, ubi-αCat, FRT40A/hsFLP; act-Gal4, α-Cat^{1}, UAS-P35/da-Gal4, UAS-GFP, α-Cat^{1}

C

en-Gal4, UAS-RFP/+ ; UAS-α-Cat-RNAi(1)/ +

en-Gal4, UAS-RFP/+ ; UAS-α-Cat-RNAi(1), α-Cat^{1}/+

D

en-Gal4, UAS-RFP/+ ; UAS-α-Cat-RNAi(1)/UAS-P35

en-Gal4, UAS-RFP/+ ; UAS-α-Cat-RNAi(1), α-Cat^{1}/UAS-P35

E

en-Gal4, UAS-RFP/+

en-Gal4, UAS-RFP/+; UAS-α-Cat-RNAi(1)/ +

en-Gal4, UAS-RFP/+; UAS-α-Cat-RNAi(1)/ UAS-P35

en-Gal4, UAS-RFP/+; UAS-α-Cat-RNAi(1), α-Cat^{1}/+

en-Gal4, UAS-RFP/+; UAS-α-Cat-RNAi(1), α-Cat^{1}/α-CatR

en-Gal4, UAS-RFP/+; UAS-α-Cat-RNAi(2)/+

en-Gal4, UAS-RFP/+; UAS-α-Cat-RNAi(2)/α-CatR

Fig 2

A

en-Gal4, UAS-RFP/+; Puc^{E697}-lacZ/+

en-Gal4, UAS-RFP/+; UAS-α-Cat-RNAi(1)/ Puc^{E697}-lacZ

en-Gal4, UAS-RFP/Rho1^{72O}/+; UAS-α-Cat-RNAi(1)/ Puc^{E697}-lacZ

en-Gal4, UAS-RFP/+; UAS-α-Cat-RNAi(1), UAS-P35/ Puc^{E697}-lacZ

C

en-Gal4, UAS-RFP, ex-lacZ/+; UAS-α-Cat-RNAi(1)/+

en-Gal4, UAS-RFP, ex-lacZ/+; UAS-α-Cat-RNAi(1), UAS-P35/+

en-Gal4, UAS-RFP/yki^{B5}

en-Gal4, UAS-RFP/yki^{B5}; UAS-α-Cat-RNAi(1)/+

D

en-Gal4, UAS-RFP/+

en-Gal4, UAS-RFP/yki^{B5}

en-Gal4, UAS-RFP/+; UAS-α-Cat-RNAi(1)/ +

en-Gal4, UAS-RFP/yki^{B5}; UAS-α-Cat-RNAi(1)/+

Fig 3

A

en-Gal4, UAS-RFP, ex-lacZ/+;

en-Gal4, UAS-RFP, ex-lacZ/UAS-DEcad-RNAi

en-Gal4, UAS-RFP, ex-lacZ/UAS-DEcad-RNAi; UAS-P35/+

B

en-Gal4, UAS-RFP/+; Puc^{E697}-lacZ/+

en-Gal4, UAS-RFP/UAS-DEcad-RNAi; Puc^{E697}-lacZ/+

en-Gal4, UAS-RFP/UAS-DEcad-RNAi; Puc^{E697}-lacZ/UAS-P35

C

en-Gal4, UAS-RFP, ex-lacZ/+

en-Gal4, UAS-RFP, ex-lacZ/UAS-DEcad-RNAi

en-Gal4, UAS-RFP, ex-lacZ/UAS-DEcad-RNAi; UAS-P35/+

D

en-Gal4, UAS-RFP/+; UAS-α-Cat-RNAi(1), α-Cat^{1}/+

en-Gal4, UAS-RFP/+; UAS-α-Cat-RNAi(1), α-Cat^{1}/UAS-DEcad::α-Cat

E

en-Gal4, UAS-RFP/+

en-Gal4, UAS-RFP/+; UAS-α-Cat-RNAi(1), α-Cat^{1}/+

en-Gal4, UAS-RFP/+; UAS-α-Cat-RNAi(1), α-Cat1/UAS-DEcad::α-Cat

Fig 4

B

en-Gal4, UAS-RFP/+; Jub::GFP/+

C

en-Gal4, UAS-RFP/+; Jub::GFP/+; UAS-α-Cat-RNAi(1)/+

D

en-Gal4, UAS-RFP/+; Jub::GFP/+; UAS-α-Cat-RNAi(1)/UAS-DEcad::α-Cat

E

en-Gal4, UAS-RFP/+; Jub::GFP/+; UAS-α-Cat-RNAi(1)/UAS-DEcadΔβ::α-CatΔN

F

en-Gal4, UAS-RFP/+

en-Gal4, UAS-RFP/+; UAS-α-Cat-RNAi(1)/+

en-Gal4, UAS-RFP/+; UAS-α-Cat-RNAi(1)/UAS-DEcad:: α-Cat

en-Gal4, UAS-RFP/+; UAS-α-Cat-RNAi(1)/UAS-DEcadΔβ::α-CatΔN

Fig 5

A

omb-Gal4/+

omb-Gal4/+; UAS-α-Cat-RNAi(1)/+

omb-Gal4/+; UAS-α-Cat

yki^{B5}/+

omb-Gal4/+; yki^{B5}/+; UAS-α-Cat/+

omb-Gal4/+; Jub-RNAi/+

omb-Gal4/+; Jub-RNAi/+; UAS-α-Cat/+

B

omb-Gal4/+

yki^{B5}/+

omb-Gal4/+; UAS-α-Cat/+

omb-Gal4/+; yki^{B5}/+; UAS-α-Cat/+

omb-Gal4/+; Jub-RNAi/+

omb-Gal4/+; Jub-RNAi/+; UAS-α-Cat/+

C

en-Gal4, UAS-RFP, ex-lacZ/+

en-Gal4, UAS-RFP, ex-lacZ/+; UAS-ft-RNAi/+

en-Gal4, UAS-RFP, ex-lacZ/+; UAS-α-Cat/+

en-Gal4, UAS-RFP, ex-lacZ/+; UAS-ft-RNAi/ UAS-α-Cat

en-Gal4, UAS-RFP, ex-lacZ/+; yki^{B5}/+; UAS-α-Cat/UAS-ft-RNAi

D

en-Gal4, UAS-RFP, ex-lacZ/+

en-Gal4, UAS-RFP, ex-lacZ/+; UAS-ft-RNAi/+

en-Gal4, UAS-RFP, ex-lacZ/+; UAS-ft-RNAi/ UAS-α-Cat

en-Gal4, UAS-RFP, ex-lacZ/+; UAS-crb-RNAi/+

en-Gal4, UAS-RFP, ex-lacZ/+; UAS-crb-RNAi/ UAS-α-Cat

en-Gal4, UAS-RFP, ex-lacZ/+; UAS-ex-RNAi/+

en-Gal4, UAS-RFP, ex-lacZ/+; UAS-ex-RNAi/UAS-α-Cat

Fig 6

A

en-Gal4, UAS-RFP/+

en-Gal4, UAS-RFP/+; UAS-α-Cat-RNAi(2)/+

en-Gal4, UAS-RFP/+; UAS-α-Cat-RNAi(2)/ UAS-α-CatR

en-Gal4, UAS-RFP/+; UAS-α-Cat-RNAi(2)/ UAS-α-CatR-ΔM

en-Gal4, UAS-RFP/+; UAS-α-Cat-RNAi(2)/ UAS-α-CatR-ΔM1

en-Gal4, UAS-RFP/+; UAS-α-Cat-RNAi(2)/ UAS-α-Cat-ΔM2

en-Gal4, UAS-RFP/+; UAS-α-Cat-RNAi(2)/ UAS-α-CatR-ΔM3

B

omb-Gal4/+

omb-Gal4/+; UAS-α-CatR-ΔM1/+

yki^{B5}/+

omb-Gal4/+; yki^{B5}/+; UAS-α-CatR-ΔM1/+

omb-Gal4/+; Jub-RNAi/+

omb-Gal4/+; Jub-RNAi/+; UAS-α-CatR-ΔM1/+

C

en-Gal4, UAS-RFP/+

en-Gal4, UAS-RFP/+; UAS-α-Cat-RNAi(2)/+

en-Gal4, UAS-RFP/+; UAS-α-Cat-RNAi(2)/ UAS-α-CatR-ΔM

en-Gal4, UAS-RFP/+; UAS-α-Cat-RNAi(2)/ UAS-α-CatR-ΔM1

en-Gal4, UAS-RFP/+; UAS-α-Cat-RNAi(2)/ UAS-α-Cat-ΔM2

en-Gal4, UAS-RFP/+; UAS-α-Cat-RNAi(2)/ UAS-α-CatR-ΔM3

D

omb-Gal4/+

omb-Gal4/+; UAS-α-CatR-ΔM1/+

yki^{B5}/+

omb-Gal4/+; yki^{B5}/+; UAS-α-CatR-ΔM1/+

omb-Gal4/+; Jub-RNAi/+

omb-Gal4/+; JubRNAi/+; UAS-α-CatR-ΔM1/+

E

en-Gal4, UAS-RFP/+

en-Gal4, UAS-RFP/+; UAS-α-Cat-RNAi(2)/ UAS-α-CatR-ΔM

F

en-Gal4, UAS-RFP, ex-LacZ/+; UAS-α-Cat-RNAi(2)/ UAS-α-CatR-ΔM

Fig 7

A

en-Gal4, UAS-RFP/+; Jub::GFP/+; UAS-α-Cat-RNAi(2)/ UAS-α-CatR

B

en-Gal4, UAS-RFP/+; Jub::GFP/+; UAS-α-Cat-RNAi(2)/ UAS-α-CatR-ΔM

C

en-Gal4, UAS-RFP/+; Jub::GFP/+; UAS-α-Cat-RNAi(2)/ UAS-α-CatR-ΔM1

D

en-Gal4, UAS-RFP/+; Jub::GFP/+; UAS-α-Cat-RNAi(2)/ UAS-α-Cat-ΔM2

E

en-Gal4, UAS-RFP/+; Jub::GFP/+; UAS-α-Cat-RNAi(2)/ UAS-α-CatR-ΔM3

F

Same genotypes as in A-E.

G

en-Gal4, UAS-RFP/+; Jub::GFP/+

vinc102.1/Y; Jub::GFP/+

H

en-Gal4, UAS-RFP/+; Jub::GFP/+

vinc102.1/Y; Jub::GFP/+

I

en-Gal4, UAS-RFP/+

vinc102.1/Y; Jub::GFP/+

Fig 8

A

en-Gal4, UAS-RFP/+; UAS-α-Cat-RNAi(2)/ UAS-α-CatR

en-Gal4, UAS-RFP/+; UAS-α-Cat-RNAi(2)/ UAS-α-CatR-H1

en-Gal4, UAS-RFP/+; UAS-α-Cat-RNAi(2)/ UAS-α-CatR-ΔβH

en-Gal4, UAS-RFP/+; UAS-α-Cat-RNAi(2)/ UAS-α-CatR-H1-ΔβH

en-Gal4, UAS-RFP/+; UAS-α-Cat-RNAi(1)/ UAS-α-CatR-3A

en-Gal4, UAS-RFP/+; UAS-α-Cat-RNAi(1)/ UAS-α-CatR-ΔABD

B

Same genotypes as in A.

C

en-Gal4, UAS-RFP/+; Jub::GFP/+; UAS-α-Cat-RNAi(2)/ UAS-α-CatR-H1

D

en-Gal4, UAS-RFP/+; Jub::GFP/+; UAS-α-Cat-RNAi(2)/ UAS-α-CatR-ΔβH

E

en-Gal4, UAS-RFP/+; Jub::GFP/+; UAS-α-Cat-RNAi(2)/ UAS-α-CatR-H1-ΔβH

F

en-Gal4, UAS-RFP/+; Jub::GFP/+; UAS-α-Cat-RNAi(2)/ UAS-α-CatR

en-Gal4, UAS-RFP/+; Jub::GFP/+; UAS-α-Cat-RNAi(2)/ UAS-α-CatR-H1

en-Gal4, UAS-RFP/+; Jub::GFP/+; UAS-α-Cat-RNAi(2)/ UAS-α-CatR-ΔβH

en-Gal4, UAS-RFP/+; Jub::GFP/+; UAS-α-Cat-RNAi(2)/ UAS-α-CatR-H1-ΔβH

Fig 9

A, B

en-Gal4, UAS-RFP/+; Jub::GFP/+; UAS-α-Cat-RNAi(2)/ UAS-α-CatR

en-Gal4, UAS-RFP/+; Jub::GFP/+; UAS-α-Cat-RNAi(2)/ UAS-α-CatR-ΔM

en-Gal4, UAS-RFP/+; Jub::GFP/+; UAS-α-Cat-RNAi(2)/ UAS-α-CatR-ΔM1

en-Gal4, UAS-RFP/+; Jub::GFP/+; UAS-α-Cat-RNAi(2)/ UAS-α-CatR-H1

C, D

Same genotypes in A.

S1 Fig

A

hsFLP; Act<CD2<GAL4, UAS-GFP/+; UAS-α-Cat-RNAi(1)/+

B

hsFLP; Act<CD2<GAL4, UAS-GFP/+; UAS-α-Cat-RNAi(2)/+

S2 Fig

Whole animal rescue plotted for following genotypes:

α-Cat^{1}

act-Gal4, da-Gal4, α-Cat^{1}/α-Cat^{1}, UAS-α-CatR

act-Gal4, da-Gal4, α-Cat^{1}/α-Cat^{1}, UAS-α-CatR-ΔM

act-Gal4, da-Gal4, UAS-DEcadΔβ::α-CatΔN

act-Gal4, da-Gal4, α-Cat^{1}/α-Cat^{1}, UAS-DEcadΔβ::α-CatΔN

S3 Fig

A

en-Gal4, UAS-RFP/+

en-Gal4, UAS-RFP/+; UAS-crb-RNAi/+

en-Gal4, UAS-RFP/+; UAS-α-Cat/+

en-Gal4, UAS-RFP/+; UAS-crb-RNAi/UAS-α-Cat

B

en-Gal4, UAS-RFP/+

en-Gal4, UAS-RFP/+; UAS-ex-RNAi/+

en-Gal4, UAS-RFP/+; UAS-α-Cat/+

en-Gal4, UAS-RFP/+; UAS-ex-RNAi/UAS-α-Cat

S4 Fig

A

en-Gal4, UAS-RFP/+

B

en-Gal4, UAS-RFP/+; UAS-α-Cat-RNAi(2)/ UAS-α-CatR

S5 Fig

en-Gal4, UAS-RFP/+; UAS-α-Cat-RNAi(2)/ UAS-α-CatR

en-Gal4, UAS-RFP/+; UAS-α-Cat-RNAi(2)/ UAS-α-CatR-ΔM

en-Gal4, UAS-RFP/+; UAS-α-Cat-RNAi(2)/ UAS-α-CatR-ΔM1
en-Gal4, UAS-RFP/+; UAS-α-Cat-RNAi(2)/ UAS-α-CatR-ΔM2
en-Gal4, UAS-RFP/+; UAS-α-Cat-RNAi(2)/ UAS-α-CatR-ΔM3
en-Gal4, UAS-RFP/+; UAS-α-Cat-RNAi(2)/ UAS-α-CatR-H1
en-Gal4, UAS-RFP/+; UAS-α-Cat-RNAi(2)/ UAS-α-CatR-ΔβH
en-Gal4, UAS-RFP/+; UAS-α-Cat-RNAi(2)/ UAS-α-CatRH1-ΔβH
en-Gal4, UAS-RFP/+; UAS-α-CatR-3A/+
en-Gal4, UAS-RFP/+; UAS-α-CatR-ΔABD/+

S6 Fig
A
omb-Gal4/+; UAS-α-CatR/+
omb-Gal4/+; UAS-α-CatR-ΔM/+
omb-Gal4/+; UAS-α-CatR-ΔM1/+
omb-Gal4/+; UAS-α-Cat-ΔM2/+
omb-Gal4/+; UAS-α-CatR-ΔM3/+
B
en-Gal4, UAS-RFP/+; UAS-α-CatR/+
en-Gal4, UAS-RFP/+; UAS-α-CatR-ΔM1/+
C
en-Gal4, UAS-RFP/+; UAS-α-CatR/+
en-Gal4, UAS-RFP/+; UAS-α-CatR-ΔM1/+
D
Same genotypes as listed in C.

S7 Fig
en-Gal4, UAS-RFP, ex-lacZ/+
en-Gal4, UAS-RFP, ex-lacZ/+; UAS-α-Cat-RNAi(2)/ UAS-α-CatR-H1
