# Supplementary material for: Role of α-Catenin and its mechanosensing properties in regulating Hippo/YAP-dependent tissue growth
Source: PLoS Genet. 2019 Nov 7;15(11):e1008454. doi: 10.1371/journal.pgen.1008454 (PMC6863567; doi:10.1371/journal.pgen.1008454)
Supplement: S2 Table — (PDF) [file pgen.1008454.s009.pdf]

S2 Table				
**Constructs**	**Amino Acids (a-Catenin)**	**Gateway cloning**	**HA tag**	**Reference**
**α-Cat Full Length**				
α-CatR	1-917	yes	yes	this work
α-Cat		no	yes	Desai et al., 2013
**DEcad-αCat Fusion Constructs**				
DEcad	0	no	no	Sarpal et al., 2012
DEcad::α-Cat	1-917	no	no	Sarpal et al., 2012
DEcadΔß::α-CatΔNT-N	281-917	no	no	this work
**M domain Constructs**				
α-CatR-ΔM	1-292 / 635-917	yes	yes	this work
α-CatR-ΔM1	1-292 / 399 -917	yes	yes	this work
α-Cat-ΔM2 (α-Cat-ΔVH2N)	1-399 / 509-917	no	yes	Desai et al., 2012
α-CatR-ΔM3	1-509 / 635-917	yes	yes	this work
**ABD Constructs**				
α-CatR-H1	683REAM > 683GSGS	yes	yes	this work
α-CatR-ΔßH	1- 811/824-917	yes	yes	Ishiyama et al., 2018
α-CatR-H1-ΔßH	1-811/824-917 + 683REAM > 683GSGS	yes	yes	this work
α-CatR-3A	L798A + I805A + V809A	yes	yes	Ishiyama et al., 2018
α-CatR-ΔABD	1-681	yes	yes	this work
